# Decidua macrophages display a restrained inflammatory profile during chorioamnionitis

**DOI:** 10.3389/fimmu.2026.1858982

**Published:** 2026-08-10

**Authors:** Neema Pithia, Carmelo V. Musumarra, Giulia Protti, Giorgia Del Vecchio, Fei-Man Hsu, Feiyang Ma, Monica Cappelletti, Tamara Tilburgs, Nina S. Prasanphanich, Lisa A. Miller, Marco Morselli, Matteo Pellegrini, Alan H. Jobe, Claire A. Chougnet, Pietro Presicce, Suhas G. Kallapur

**Affiliations:** 1 Divisions of Neonatology and Developmental Biology, David Geffen School of Medicine, University of California, Los Angeles, Los Angeles, United States; 2 Department of Chemistry, Life Sciences and Environmental Sustainability (S.C.V.S.A.), University of Parma, Parma, Italy; 3 Department of Biotechnology and Biosciences, University of Milano-Bicocca, Milan, Italy; 4 Institute for Quantitative and Computational Biosciences–Collaboratory, University of California, Los Angeles, Los Angeles, United States; 5 Department of Pathology and Laboratory Medicine, UCLA Immunogenetics Center, Los Angeles, CA, United States; 6 Division of Immunobiology, Cincinnati Children’s Hospital Research Foundation, The University of Cincinnati College of Medicine, Cincinnati, OH, United States; 7 Department of Pediatrics, University of Cincinnati College of Medicine, Cincinnati, OH, United States; 8 Division of Infectious Disease, Cincinnati Children’s Hospital Medical Center, Cincinnati, OH, United States; 9 California National Primate Research Center, University of California, Davis, Davis, United States; 10 Department of Molecular, Cell and Developmental Biology Medicine, University of California, Los Angeles, Los Angeles, United States; 11 Division of Neonatology/Pulmonary Biology, Cincinnati Children’s Hospital Research Foundation, and the University of Cincinnati College of Medicine, Cincinnati, OH, United States

**Keywords:** chorioamnionitis, inflammation, reproductive immunology, innate immunity, macrophages

## Abstract

**Introduction:**

Decidual macrophages (DMs) are strategically located at the maternal-fetal interface to orchestrate innate host defense while balancing tolerance to the allogenic fetus. An important pregnancy complication is chorioamnionitis characterized by infection/inflammation in the fetal membranes and the amniotic fluid with upregulation of TNFα and other pro-inflammatory cytokines. The goal of this study was to determine maternal vs fetal origin of DMs and to investigate whether TNF signaling play a role in DM response to chorioamnionitis.

**Methods:**

Decidua tissue from pregnant Rhesus macaques given intraamniotic injection of lipopolysaccharide/saline with or without the TNF inhibitor Adalimumab (n=33) and human subjects with/out chorioamnionitis (n=11) were used for bulk RNAseq, scRNAseq, and flow cytometry experiments.

**Results:**

In both human and Rhesus macaques, DMs coordinately upregulate both pro- and anti-inflammatory cytokines during chorioamnionitis. Although DMs did not express TNF during chorioamnionitis, inhibition of TNF signaling downregulated 50% of LPS induced genes. About 4% of the DMs were of male fetus origin. These fetal origin DMs selectively upregulated *IL6* mRNA during chorioamnionitis, suggesting a potential role in fetal immune priming.

**Discussion:**

We suggest that DMs orchestrate a carefully balanced host defense with a restrained pro-inflammatory profile during chorioamnionitis to potentially prevent adverse outcomes while protecting the mother-fetus dyad. Activation of fetal origin macrophages during chorioamnionitis may have implications for shaping neonatal immune responses.

## 1 Introduction

Tissue resident macrophages display niche and context specific functions adapting their identity and activity to fulfill distinct physiological roles essential for tissue-specific homeostasis, immune responses, and repair (1). At the maternal-fetal interface, tissue resident macrophages are abundant and account for 20-30% of immune cells within the decidua (2). While early paradigms frequently classified tissue-resident macrophages into binary M1 or M2 activation states, subsequent research has revealed an intricate, multi-layered landscape of decidual macrophage (DM) heterogeneity (3). Thus, DMs play critically important roles in balancing fetal tolerance with host defense and tissue remodeling to ensure successful pregnancy outcomes (4). These baseline phenotypes are dynamically regulated by localized microenvironmental factors native to the decidua (5).

Acute chorioamnionitis, an important pregnancy complication, is caused by microorganisms ascending from the lower genital tract, and is characterized by neutrophil driven inflammation at the maternal-fetal interface (2). However, there is a paucity of data regarding DM phenotypes during chorioamnionitis. This is an important question since chorioamnionitis presents a fundamental challenge to the pregnant mother in balancing host defense with fetal tolerance.

To better understand the immunobiology, we modeled chorioamnionitis in the Rhesus macaque which shares similar reproductive immunology and pregnancy physiology with humans. We previously reported that intraamniotic injection of LPS closely simulates human chorioamnionitis (6). In the LPS model, neutrophils are a prime-driver of inflammation at the maternal-fetal interface (7). Furthermore, LPS responses and neutrophil driven inflammation were significantly attenuated by blocking TNF signaling (7).

In the present study, we utilized the same Rhesus macaque LPS model to understand DM function during chorioamnionitis. Since our proof-of-concept experiments were aimed at understanding the role of TNF signaling, an anti-TNFα antibody Adalimumab (Adal) was given prior to LPS. To establish clinical relevance, human placentae from preterm deliveries with or without histologic diagnosis of chorioamnionitis were used. DM immunobiology was studied using bulk and scRNAseq, flow cytometry, and functional studies. Because our objective was to define the host response to inflammatory challenge, we prioritized macrophage pro- and anti-inflammatory cytokine expression as key functional outputs. Since prior studies classified macrophage polarization states as M1 (Th1-like cytokines) or M2 (Th2-like cytokines) (8), we analyzed the M1/M2 profile of DMs in different exposure conditions.

We report that both in the Rhesus macaque and humans, a significant proportion of third trimester DMs express both M1 and M2 markers that do not significantly change during chorioamnionitis. Both pro- and anti-inflammatory cytokines are upregulated in the DMs during chorioamnionitis. Approximately 3-4% of DMs are of fetal origin, and they are responsive to inflammatory stimuli. Surprisingly, DMs do not significantly express TNF and many other classic pro-inflammatory cytokines during chorioamnionitis. However, inhibition of TNF signaling in the DM inhibited about 50% of LPS induced genes. These results suggest a restrained pro-inflammatory profile of DM during chorioamnionitis.

## 2 Results

Chorioamnionitis was induced in pregnant Rhesus macaques with intraamniotic (IA) injection of lipopolysaccharide (LPS). Women delivering preterm with or without histologically confirmed chorioamnionitis were recruited (**Supplementary Figure S1A, B** shows the experimental groups and study design). IA LPS increased DM cell counts modestly ~2-fold compared to Controls. TNFα inhibitor (Adalimumab) injected prior to LPS to completely inhibit TNF signaling (Adal+LPS) decreased DM counts to control levels in the Rhesus (**Supplementary Figure S2**). However, in human samples, there was no significant difference in DM counts between chorioamnionitis negative (Chorio -) and chorioamnionitis positive (Chorio +) subjects (**Supplementary Figure S2**).

### 2.1 Bulk RNAseq reveals that both pro- and anti-inflammatory pathways are activated in Rhesus macaque decidua macrophages after LPS-exposure

**Figure 1:**
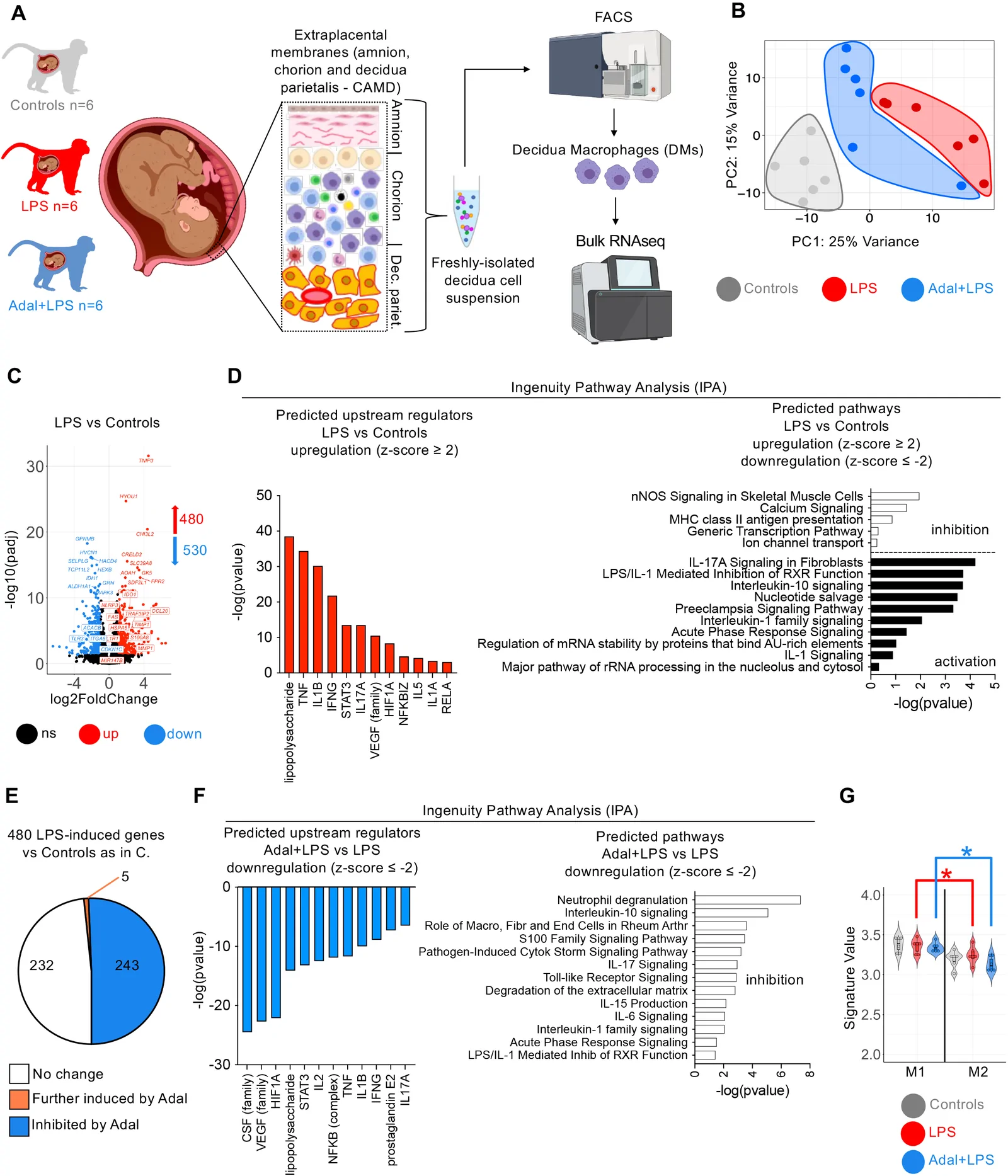
Intrauterine inflammation changes the global transcriptomic landscape of Rhesus decidua macrophages (DMs) in a partially TNF-dependent manner. Schematic of the groups (See **Supplementary Figure S1A** for the experimental design). Extraplacental membranes (amnion, chorion, and decidua parietalis) were dissected free of placenta after delivery and a cell suspension of decidua parietalis was created. Decidua macrophages (DMs), defined as CD45^+^CD3^-^CD19^-^CD20^-^CD56^-^CD14^high^CD88^+^HLA-DR^+^ cells, were flow-sorted [>98% macrophage purity (**Supplementary Figure S3**)], and used for bulk RNAseq. **(B)** Principal-component analysis (PCA) of DMs in Rhesus Controls (grey) vs LPS (red) exposed animals represents distinct clustering. The PCA shows an intermediate clustering of Adal+LPS (blue) group. **(C)** The volcano plot displays differentially expressed genes (DEGs) in LPS vs Control with blue dots being downregulated (530 genes) and red upregulated (480 genes) (DEG≥2-fold change, FDR adjusted p value ≤ 0.05, baseMean>10). **(D)** Bar graphs show the top predicted upstream regulators and predicted activated/inhibited pathways of genes induced by LPS vs Control ranked by p value on Ingenuity Pathway Analysis (IPA). **(E)** Pie chart showing 480 genes induced by LPS as in **(C)** Of those 480 genes, 243 genes are downregulated by Adalimumab (↓ ≥1.5-fold change vs. LPS), 232 do not change their expression upon Adalimumab treatment (↓1.5 < x < ↑1.5-fold change vs. LPS), and 5 genes are upregulated by Adalimumab (≥↑1.5-fold change vs. LPS). (FDR adjusted p value ≤ 0.05, FC 2 (log2FC 1), baseMean > 10). **(F)** IPA shows the predicted upstream regulators and the pathways inhibited by Adalimumab treatment ranked by p value. **(G)** Violin plots of module score illustrate the analysis of macrophage polarization states within DMs of different groups. (*p<0.05 two-way ANOVA).

We asked if the transcriptomic profile of DM changed during chorioamnionitis and whether TNF signaling played a role. We initially used a bulk RNAseq approach, which allows robust analyses using large numbers of cells with a high sequencing depth. The groups were Controls (n=6), LPS (n=6), and Adal+LPS (n=6) (**Figure 1A**; **Supplementary Tables 1A, B**). Flow cytometry-sorted DMs defined by well-established canonical markers of macrophages were used and the post-sorting purity was verified by morphologic evaluation (**Supplementary Figure S3**). Principal component analysis (PCA) revealed distinct global transcriptional signatures for the groups Controls vs. LPS (**Figure 1B**). Interestingly, Adal+LPS group exhibited an intermediate transcription profile between Controls and LPS (**Figure 1B**). Compared to Controls, LPS upregulated 480 and downregulated 530 differentially expressed genes (DEGs) (**Figure 1C**; **Supplementary Table 2**). Inspection of the LPS-induced upregulated genes revealed both pro-inflammatory and anti-inflammatory genes (**Supplementary Table 2**). Among the pro-inflammatory genes were *S100A8, S100A9, S100P, CXCL1, CXCL13, CCL2, CCL20, PPBP (CXCL7), CSF3*, predicting innate immune activation, recruitment of neutrophils, and amplification of inflammatory response. NF-κB, TLR/IL1, and inflammasome pathway appeared to mediate inflammatory responses as evidenced by increased expression of *NLRP3, NLRP12, IL1R1, IL1RAP* genes. On the other hand, several anti-inflammatory genes were also upregulated by LPS. These include *IL1RN, TNIP3, TRAF1, PDL1, SLC39A8, IDO1, IDO2, ARG2, AOAH, DOK3, FPR2*, and *SIGLEC10*. Some of these genes are known to be downstream of NF-κB, TNF, and IL1 signaling. Interestingly, several micro-RNAs known to be anti-inflammatory were also upregulated by LPS. These include *mIR147b*, *mIR233*, and *mIR590*. Both pro-apoptotic (*FAS, ANXA3*) and anti-apoptotic (*TRAF3IP2*) were induced by LPS.

Inspection of LPS downregulated genes revealed several genes involved in TLR signaling and pro-inflammation including *TLR3, TLR5, TLR7, TLR8, CXCR4, MPO, CD68, ALPK1*, and *LTA4H* (**Figure 1C**; **Supplementary Table 2**). On the other hand, the anti-inflammatory gene *TGFB2* was also downregulated by LPS. Other notable functional categories downregulated by LPS included lipid and steroid metabolism genes (*ACACB, ACAD10, ACOX2, ALDH1A1, CYP19A1, FATP4*), cell adhesion (*ITGA6, CADM1*), and cell cycle regulation (*CDKN1C, CCNG2, CDCA2*).

The predicted upstream regulators of LPS upregulated genes in DMs included classic pro-inflammatory cytokine pathways including *TNF, IL1, IFNG, IL17A, STAT3, HIF1A, RELA*, but also included anti-inflammatory pathways such as *VEGF, NFKBIZ, IL5* (**Figure 1D**). Consistently, Ingenuity Pathway Analysis (IPA) revealed LPS upregulated pathways to include pro-inflammatory IL1 and IL17A signaling and anti-inflammatory IL10 signaling (**Figure 1D**). LPS downregulated pathways included MHCII antigen presentation and ion/Calcium transport (**Figure 1D**).

### 2.2 TNF signaling partially mediates Rhesus macaque decidua macrophage responses to LPS

Next, we asked if these changes in gene expression induced by LPS are downstream of TNF signaling. Adalimumab treatment downregulated over 50% (243 out of 480) of these LPS-induced genes indicating that TNF pathway is an important mediator of LPS responses in the DMs (**Figure 1E**; **Supplementary Table 3**). Ingenuity pathway analysis of these downregulated genes showed substantial overlap with the LPS-induced upstream regulators and pathways (**Figure 1F**), reinforcing the role of TNF signaling in driving LPS-induced inflammatory response in DMs.

### 2.3 Rhesus macaque decidua macrophages exhibit a hybrid M1/M2 polarization state

Given the mixed regulation of both pro- and anti-inflammatory genes, we comprehensively assessed macrophage activation states using gene signature (module) scoring based on curated M1 and M2 gene sets applied to bulk RNAseq data (**Supplementary Table 4**). M1 and M2 signature values were similar across all exposure groups (**Figure 1G**), although there was a slight skewing towards a higher M1 vs M2 signature value for LPS and Adal+LPS groups (**Figure 1G**). Overall, these results suggest that DM transcriptional profiles do not conform to a polarized classical M1 or M2 activation pattern.

### 2.4 Transcriptomic profiling at single-cell resolution reveals mixed pro- and anti-inflammatory decidua macrophages following LPS in the Rhesus macaques

**Figure 2:**
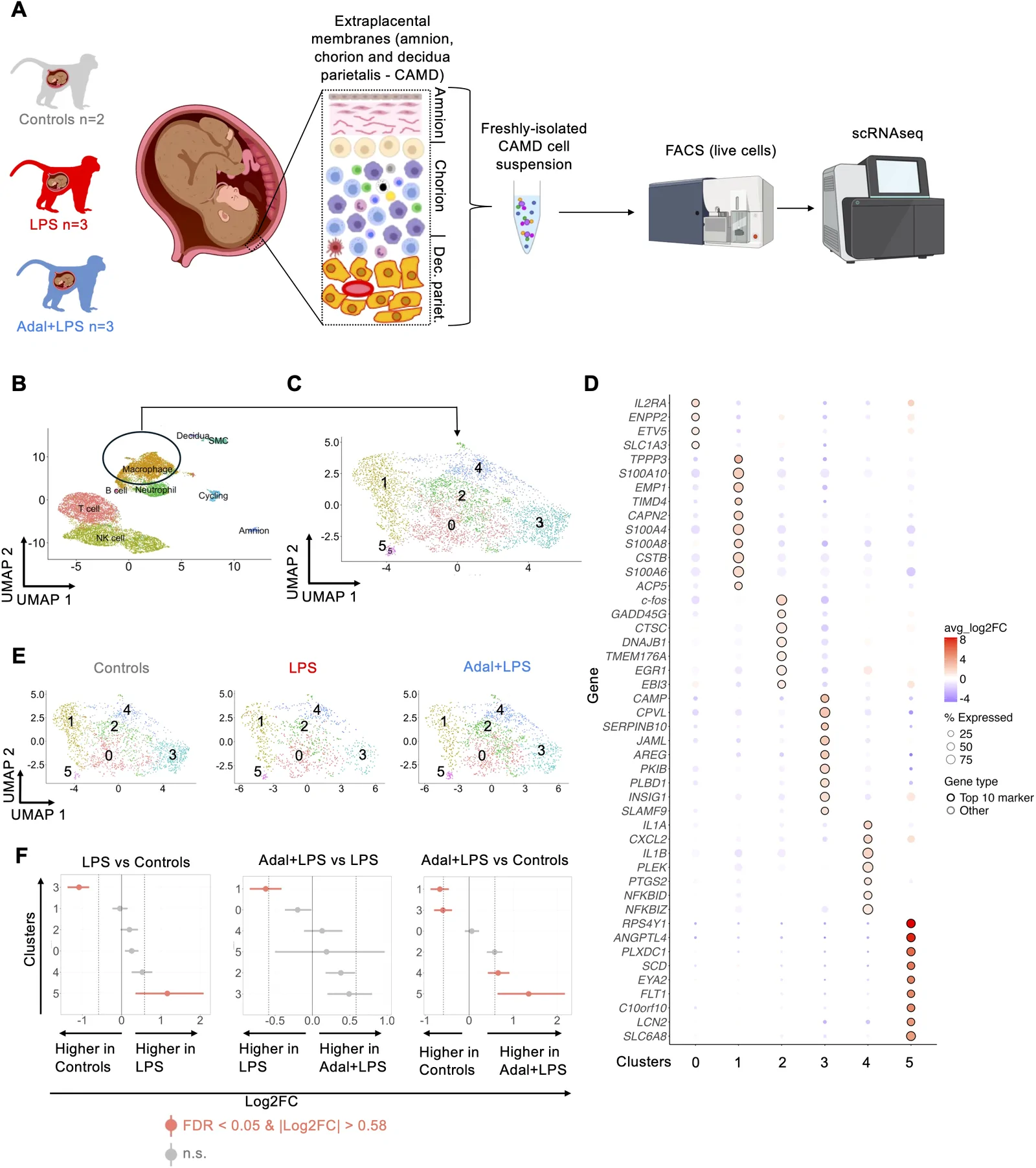
scRNAseq demonstrates that intraamniotic LPS induces both pro- and anti- inflammatory markers in Rhesus macaque DMs. **(A)** Schematic of the groups. Extraplacental membranes (amnion, chorion, and decidua parietalis) were dissected free of placenta and cell suspensions were created. Live cells were flow-sorted with Sytox green and scRNAseq was performed. **(B)** Uniform Manifold Approximation and Projection (UMAP) plot shows Rhesus cells colored by cell types and the decidua macrophage (DM) cluster is circled. **(C)** UMAP plot depicts six Rhesus DM clusters by unsupervised method. **(D)** Dot plot showing the top representative marker genes for each DM cluster. **(E)** UMAP plots show DM clusters based on exposures. **(F)** Relative abundance of different clusters. Significant differences in cell proportions between conditions are indicated by a red line (FDR < 0.05, |Log 2 FC| > 0.58).

To further understand the cell level transcriptomic changes, we performed single cell RNA seq (scRNAseq) on dissociated fetal membranes (chorion-amnion-decidua parietalis) from the three groups (Control n=2; LPS n=3, Adal+LPS n=3) (**Figure 2A**). Of note, 5/8 donors used for scRNAseq were also included in the bulk RNAseq dataset (**Supplementary Table 1B**). Major cell types in the fetal membranes including immune and non-immune cells were identified (**Figure 2B**). Macrophages were identified from other cell populations using annotation based on marker genes from published studies (9–13) (**Figure 2B**). Re-clustering to resolve DM heterogeneity revealed six DM clusters by uniform manifold approximation and projection (UMAP) (**Figure 2C**). Inspection of the top 10 cluster marker genes revealed that known pro-inflammatory genes were expressed in different clusters (**Figure 2D**; **Supplementary Table 5**): For e.g. *IL2RA* (CD25) in cluster 0, and *IL1A*, *IL1B* and *CXCL2* in cluster 4. Conversely, many subsets had both pro- and anti-inflammatory genes represented: For e.g. cluster 4 had high levels of expression of pro-inflammatory genes (*IL1A, IL1B, PTGS2*) but also has high levels of expression for inflammation dampening genes in the NF-κB pathway (*NFKBID, NFKBIZ*). Each cluster was represented in all prenatal exposure groups, but the distribution revealed an overrepresentation of cluster 3 DMs in Controls and cluster 5 DMs after LPS. Cluster 1 DMs seemed to be responsive to TNF inhibition as the relative abundance of cluster 1 DMs was the lowest in Adal+LPS DMs (**Figures 2E, F**). Interestingly, cluster 1 includes several genes from the *S100* family involved in innate immune inflammation (**Figure 2D**). Cluster 3 marker genes include genes serving innate immunity (*CAMP* encoding LL37) and gene encoding the protease inhibitor SERPINB10 which is a known M2 macrophage marker (**Figure 2D**). Many genes in cluster 4 encode pro-inflammatory cytokines including *IL1A, IL1B, PTGS2* (Cox2) (**Figure 2D**). Cluster 5 had high levels of expression of *RPS4Y1*, a gene encoded in the Y-chromosome, indicating fetal origin of DM represented in this subset (**Figure 2D**). Similar to bulk RNAseq, the unsupervised clustering of DMs by scRNAseq did not confirm distinct pro- or anti-inflammatory function or M1/M2 polarization.

Compared to Controls, LPS-exposure differentially induced 93 upregulated and 45 downregulated transcripts across all clusters (**Supplementary Figure S4**; **Supplementary Table 6**). Thirty four out of the 93 upregulated genes were common to both the scRNAseq and bulk RNAseq data sets (**Supplementary Figure S5**). Inspection of the common LPS-induced genes revealed both pro-inflammatory and anti-inflammatory genes. LPS upregulated genes included pro-inflammatory (*S100A9, CXCL13, CCL2, CCL8*) and anti-inflammatory genes (*TNIP3, TRAF1, PDL1* (CD274)*, SLC39A8, IDO1, AOAH, DOK3, FPR2*). Sixteen out of 45 downregulated genes in the DM scRNAseq were common to bulk RNAseq dataset (**Supplementary Figure S5**). Many common genes were pro-inflammatory (M1) particularly in the leukotriene pathway (*CYSLTR1*), but some also were previously shown to promote M2 polarization of macrophage (*HGF, PLXDC1*) (14). Taken together, these data suggest that at a single cell resolution, rhesus macaque DMs do not segregate transcriptionally as being pro/anti-inflammatory or by M1/M2 profile.

### 2.5 Single-Cell profiling reveals mixed pro- and anti-inflammatory macrophage activation in human decidua macrophages during chorioamnionitis

**Figure 3:**
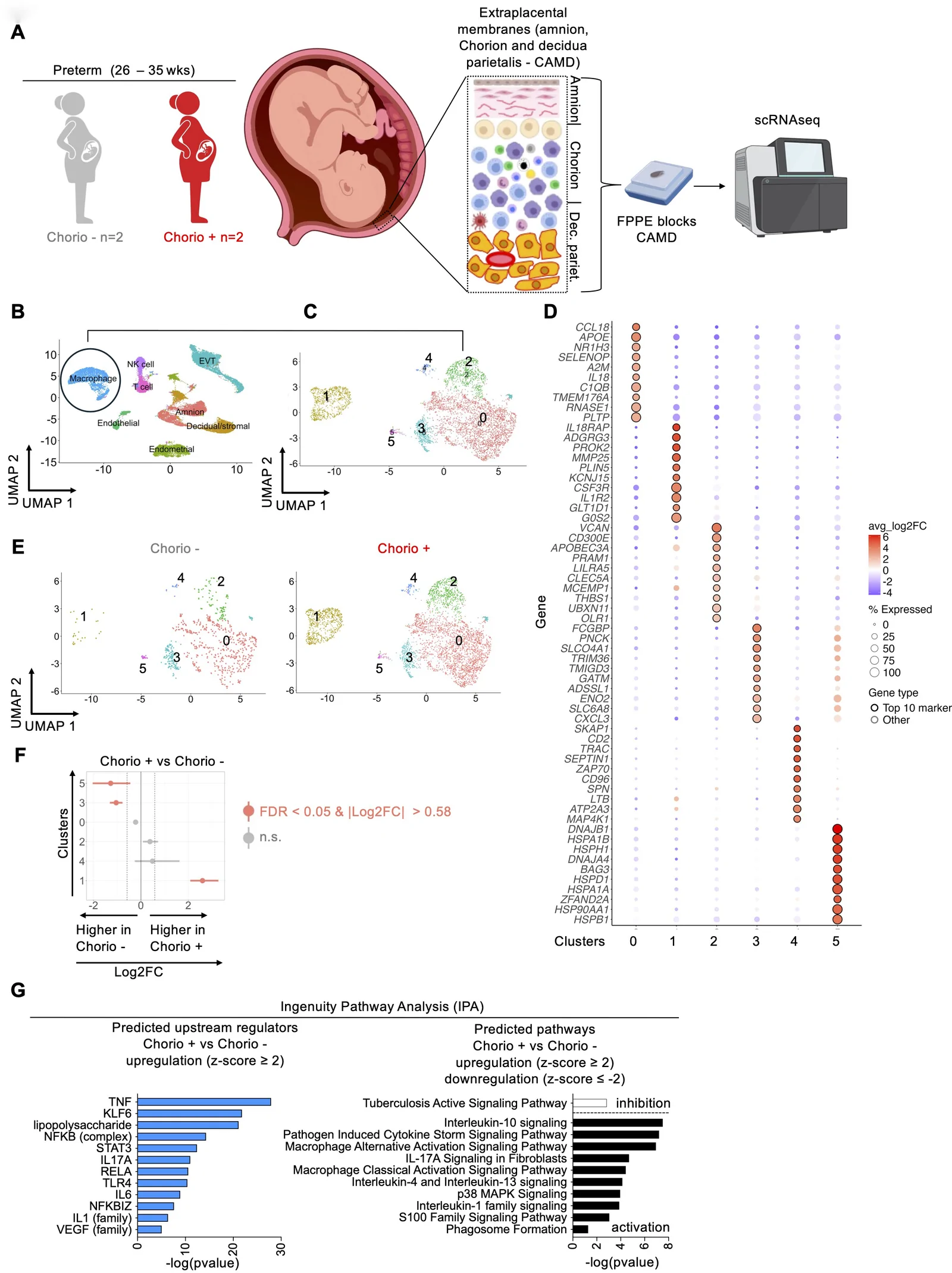
Chorioamnionitis increases both pro- and anti-inflammatory genes in multiple human DM clusters. **(A)** Schematic of the experiment is shown. Extraplacental membranes (amnion, chorion, decidua parietalis) were removed after birth, formalin fixed and paraffin embedded (FFPE) blocks were generated. Sections were cut and slices were rehydrated followed by creation of cell suspensions for scRNAseq. **(B)** UMAP plot shows cell types. DM cluster is circled. **(C)** UMAP of DM depicts six human DM clusters by unsupervised method. **(D)** Dot plot showing the top representative marker genes for each human macrophage cluster. **(E)** UMAP plots show human DM clusters based on the diagnosis of chorioamnionitis. **(F)** Relative abundance of different clusters. Significant differences in cell proportions between conditions are indicated by a red line (FDR < 0.05, |Log 2 FC| > 0.58). **(G)** IPA shows the top predicted upstream regulators and predicted activated/inhibited pathways of genes induced by chorioamnionitis ranked by p value.

To understand if the findings from Rhesus macaque chorioamnionitis induced by LPS are generalizable and relevant to human pathology, we performed scRNAseq using four human fetal membranes from donors delivering at preterm gestation (Chorio + n=2, Chorio – n=2). (**Figure 3A**; **Supplementary Tables 7A, B**). Single cells were isolated from formalin fixed paraffin embedded extraplacental chorion-amnion-decidua sections as previously described (10). Major cell types including immune and non-immune cells were identified with the exception of neutrophils which are underrepresented due to known assay related loss (**Figure 3B**). Macrophages were re-clustered and six distinct DM clusters were identified on UMAP visualization (**Figure 3C**). Similar to the Rhesus, human scRNAseq data showed DMs with pro-inflammatory genes being represented in different clusters (*IL18* in cluster 0, *IL18RAP* in cluster 1, *CXCL3* in cluster 3) (**Figure 3D**; **Supplementary Table 8**). Since cluster 1 occupied a distinct UMAP space and had genes that are also expressed by neutrophils, we analyzed the expression profiles of lineage-specific markers. Notably, cluster 1 showed a robust expression of *ITGAX* (CD11c) and *CD14* with no/minimal expression of neutrophil-specific genes such as *ELANE* (neutrophil elastase) and *CEACAM8* (CD66b) (**Supplementary Figure S6**), suggesting a monocyte/macrophage lineage of cluster 1. Interestingly, cluster 1 DMs was overrepresented in Chorio + samples (**Figures 3E, F**), suggesting recruitment of activated monocyte/macrophages during chorioamnionitis. Cluster 3 and cluster 5 DMs were overrepresented in Chorio - samples (**Figures 3E, F**). Another theme common with Rhesus macaque was that both pro- and anti-inflammatory genes were expressed in the same cluster (*IL18* and *APOE* in cluster 0, *CSF3* and the IL1 decoy receptor *IL1R2* in cluster 1, *CXCL3* and the M2 marker *ENO2* in cluster 3). The small cluster 5 had many genes in the heat-shock protein pathway (*HSPA1A, HSPA1B, HSPD1, HSPH1*, and *HSPB1*) indicating activation of stress-response or function as an alarmin (**Figure 3D**). Compared to Chorio negative subjects, chorioamnionitis exposure differentially upregulated 44 and downregulated 10 transcripts (**Supplementary Figure S7**; **Supplementary Table 9**). Upregulated genes include members of TNF receptor superfamily such as *TNFRSF18* (GITR), *TNFRSF4* (OX40), *TNFRSF9* (4-1BB), and *TNFRSF8* (CD30) which encode co-stimulatory receptors that potentiate NF-κB and MAPK-dependent signaling. Similar to Rhesus, scRNAseq analysis in human samples also revealed both pro- and anti-inflammatory genes upon chorioamnionitis exposure such as *IL1R1* and *IL1R2*, respectively. Ten genes were downregulated in DM upon chorioamnionitis (**Supplementary Figure S7**; **Supplementary Table S9**): *PER3, IFI44L, ATP1B1, TNFSF18(GITRL), HPGDS, FGL1, LAG3, GATM, CCDC194*, and *USP18*, suggesting roles associated with homeostatic, regulatory, or anti-inflammatory macrophage states. Similar to the Rhesus macaque, the predicted upstream regulators of chorioamnionitis upregulated genes in DMs included classic pro-inflammatory cytokine pathways and M1 polarization including (*TNF, IL1, IL6, STAT3, RELA*) but also included anti-inflammatory pathways and M2 polarization including (*VEGF, NFKBIZ*) (**Figure 3G**). Ingenuity pathway analysis revealed chorioamnionitis upregulated pathways to include pro-inflammatory IL1 and IL17A signaling and anti-inflammatory IL10 signaling (**Figure 3G**).

To further understand if the cluster level co-expression of M1/M2 genes also occurs at an individual cell level, we evaluated expression of well-established pro- and anti-inflammatory genes. scRNAseq analysis showed the co-expression of M1 and M2 marker genes in DM of both Rhesus and human, regardless of the treatment or presence of inflammation (**Supplementary Figure S8**). Consistent with results from Rhesus macaques, human individual cell data confirm cluster level inferences that both M1 and M2 markers are co-expressed in the DMs.

### 2.6 Common genes in Rhesus and human induced by chorioamnionitis reveal a mixed pro- and anti-inflammatory role for DMs

**Figure 4:**
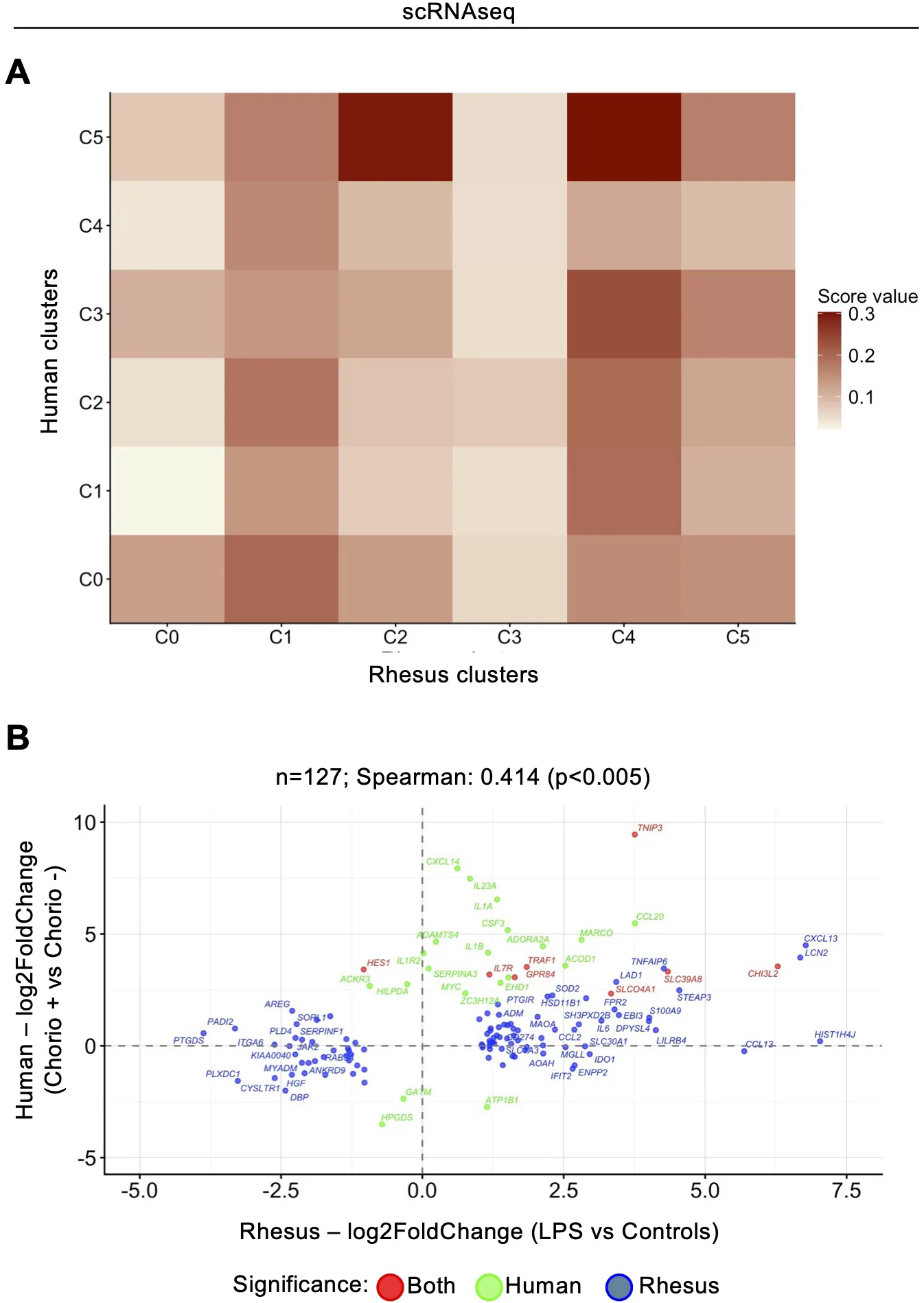
Rhesus and human DMs express common differentially expressed genes during chorioamnionitis. **(A)** Heatmap showing the similarity score between Rhesus and human DM clusters, calculated using the AddModuleScore_UCell function. **(B)** Log scale X-Y plot of differentially expressed DM genes (DEG) in the Rhesus (x-axis) vs human (y-axis). Spearman correlation of 0.41 between the two indicates moderate correlation (p<0.005). Seven upregulated common genes have both predicted pro- and anti-inflammatory functions.

We sought to determine whether DMs in Rhesus macaques and humans share gene signatures. Cross-species similarity between DMs was observed, as indicated by moderate to high similarity scores in most clusters (**Figure 4A**; **Supplementary Table 10**). Furthermore, we reasoned that pseudobulk analysis of differentially expressed genes that are common to both human and Rhesus macaque chorioamnionitis will provide a more robust and generalizable understanding of the role of DMs in this process. Most of the differentially expressed genes during chorioamnionitis in the Rhesus (blue) and human (green) were located in the right upper (common upregulated) or left lower quadrant (common downregulated) (**Figure 4B**). Indeed, the Spearman’s correlation between the two data sets was 0.41 indicating a moderately good correlation (p<0.005). Of the common upregulated genes between Rhesus macaque and human DMs (shown in red in **Figure 4B**), seven genes were upregulated by ≥ 2-fold with an adjusted p-value ≤0.05. These seven common genes represent both pro-inflammatory (*TRAF1, IL7R* (CD127)) (15), and anti-inflammatory function (*SLC39A8/ZIP8*, *TNIP3*, both NF-kB inhibitors) (16). *CHI3L2*, a chitinase like protein, is involved in tissue remodeling and is known to be a M2 marker (17). Taken together, these data suggest that during chorioamnionitis DMs orchestrate an innate immune response while simultaneously engaging NF-κB brakes to dampen inflammation.

### 2.7 Flow cytometry of decidua macrophages demonstrate a hybrid M1/M2 polarization state

**Figure 5:**
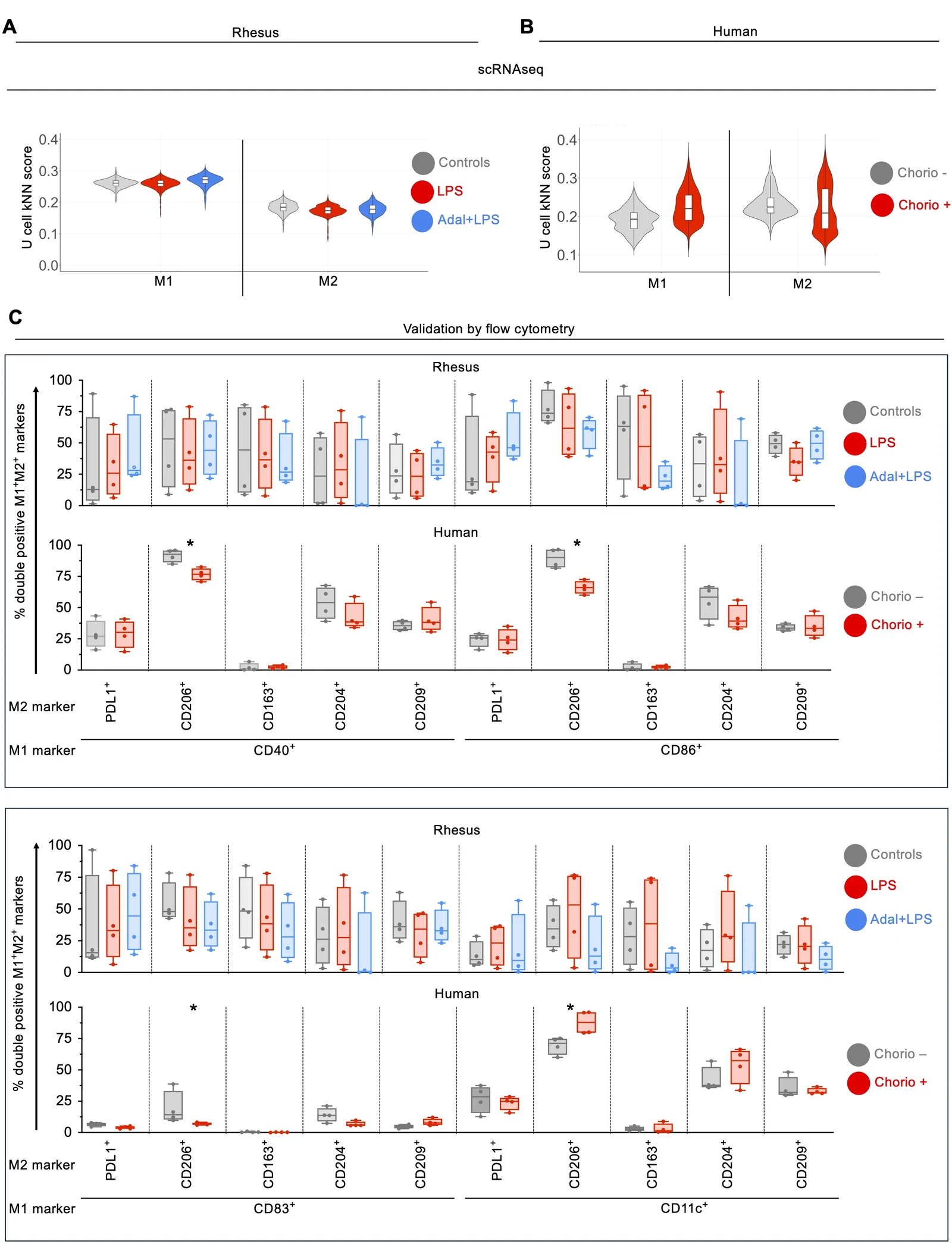
Rhesus and human DMs co-express M1 and M2 markers that do not change during chorioamnionitis. **(A, B)** Violin plots of module scores show a similar U cell kNN score for M1 and M2 markers regardless of exposures in Rhesus and the presence of chorioamnionitis in human. **(C)**. Flow cytometry of decidua cell suspensions stained with a cocktail of M1 (CD11c, CD83, CD86, and CD40) and M2 (CD206, CD163, PDL-1[CD274], CD209, and CD204) markers. Percent of double-positive populations with M1 and M2 markers. The frequency does not change with exposures in the Rhesus macaque. In human DMs only CD206 and CD11c frequency is different based on chorioamnionitis exposures. (*p < 0.05, Mann Whitney U-test).

To further validate hybrid DM polarization states, we analyzed scRNAseq datasets (**Supplementary Tables 5**, **S8**) with validation by flow cytometry using well-established macrophage M1/M2 markers (**Supplementary Table 11B**) (18–20). Due to sparsity of gene signatures in scRNAseq datasets, we used U cell scores with k-Nearest Neighbor smoothing (kNN) to robustly infer M1/M2 gene signatures. U cell kNN scores for M1 and M2 states did not change after exposure to chorioamnionitis in both Rhesus and human DM scRNAseq datasets (**Figures 5A, B**). However, Rhesus DMs had a higher M1 compared to M2 value for all groups, while the scores were similar in human DMs. These data further reinforce the results from bulk RNAseq that DMs do not change M1/M2 polarization during chorioamnionitis.

Next, we performed multi-parameter flow cytometry on dissociated decidua cells from both human and Rhesus macaques using a panel comprising well-established M1 and M2 cell surface markers (**Figure 5C**; **Supplementary Figures S9A, B**; **Supplementary Table 11B**). Most of the markers tested were expressed by both Rhesus macaque and human DMs except minimal expression of CD163 (M2 marker) in human (**Supplementary Figures S9A, B**). Overall, M1 and M2 markers were co-expressed in a large number of DMs in both species. Furthermore, there was no significant shift in the pattern of co-expression during chorioamnionitis in the Rhesus macaques (**Figure 5C**; **Supplementary Figure S9A**). In the human, DMs from human donors with chorioamnionitis had significantly higher CD206^+^CD11c^+^, but lower CD206^+^CD83^+^ expression compared to Chorio negative subjects (**Figure 5C**; **Supplementary Figure S9B**). These flow cytometry data powerfully reinforce the conclusions from both bulk RNAseq and scRNAseq data that DMs exhibit a hybrid polarization state, and further DM polarization does not shift significantly during chorioamnionitis.

### 2.8 Decidua macrophages are not the major source of TNF during acute chorioamnionitis

**Figure 6:**
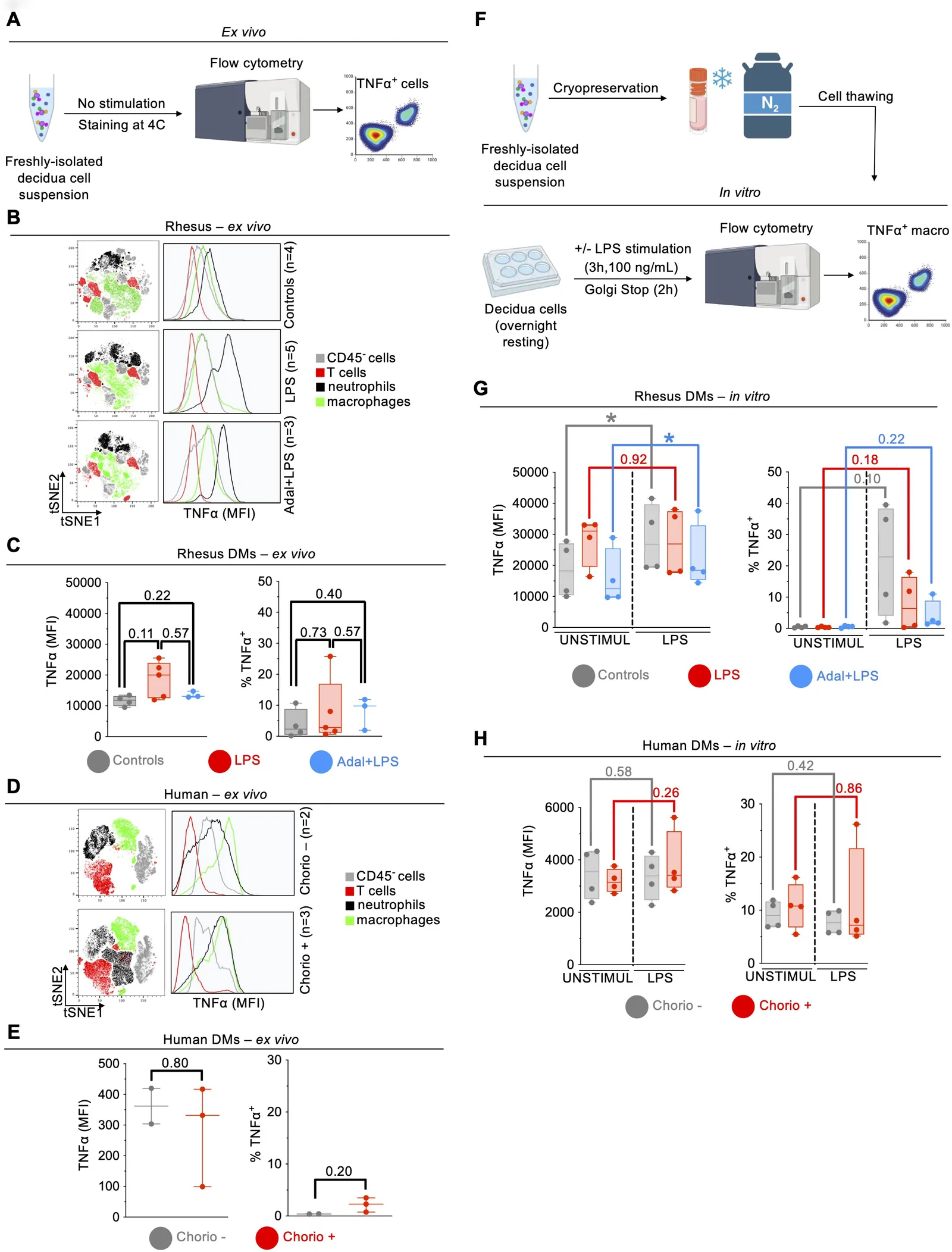
DMs do not express TNFα during chorioamnionitis with a variable *in vitro* responsiveness to LPS. TNFα production was assessed both *ex vivo* and *in vitro* by the decidua cells. **(A–E)** represent *ex vivo* analysis. **(A)** Rhesus decidua cells isolated immediately post-delivery without exogenous stimulation were stained with a cocktail of surface mAbs for the different immune/non-immune cells and intracellular TNFα expression was evaluated in DMs by flow cytometry. **(B)** High dimensional flow cytometry Rhesus data including all parameters (fluorescence channels) for pooled samples were concatenated by exposure conditions (Controls n=4; LPS n=5; Adal+LPS n=3). Data were visualized by dowsampling and reducing to two-dimension tSNE plots. Composite histogram overlay displaying TNFα MFI expression across the four gated populations for different decidua cell types (CD45^-^ cells (grey), T cells (red), neutrophils (black), and macrophages (green)). Compared to controls, neutrophils exposed to LPS had a higher MFI for TNFα. Compared to LPS, neutrophils from Adal+LPS group had a lower TNFα MFI. For macrophages, T cells, and CD45 negative cells, TNFα MFI did not change between exposure conditions. **(C)** There were no significant differences in the MFI and frequency of TNFα^+^ cells in the Rhesus DMs across the treatments. **(D)** Human decidua tSNE plots were generated as in B (Chorio - n=2; Chorio + n=3). Decidua neutrophils TNFα MFI from Chorio + group showed a minimal increase. For macrophages, T cells, and CD45 negative cells TNFα MFI did not change. **(F–H)** represent *in vitro* analysis. **(F)** Decidua cells including immune cells and non-immune cells were isolated by enzymatic digestion and immediately cryopreserved. Prior to the *in vitro* studies, decidua cells were thawed and cultured in plates for overnight resting/recovery followed by LPS challenge (100 ng/mL for 3h). After 1h, Golgi stop was added and 2h later the cells were stained with a cocktail of surface mAbs for the different immune/non-immune cells and intracellular TNFα expression was evaluated in DMs by flow cytometry. **(G)** In the Rhesus, unstimulated DMs did not produce TNFα in all the experimental groups. Upon *in vitro* LPS challenge, Controls and Adal+LPS DMs (but not LPS DMs) significantly increase the MFI of TNFα DMs. **(H)** In human, DMs minimal expression of TNFα was detected at baseline and *in vitro* LPS stimulation did not increase the TNFα production. TNFα^+^ DMs are shown as mean ± SEM (*p < 0.05, paired t test).

Bulk RNAseq data in the Rhesus did not show LPS-induced upregulation of *TNF* and other key pro-inflammatory cytokines in the DMs (**Supplementary Figure S10**). In both Rhesus and human scRNAseq datasets low *TNF* mRNA expression was noted confirming the bulk RNA seq data (**Supplementary Figures S11A, B**). To understand expression of TNFα protein, we reanalyzed flow cytometry data of TNFα production previously obtained from *ex vivo* Rhesus decidua cells isolated immediately post-delivery without exogenous stimulation (**Figure 6A**) (6). Dissociated decidua cells were used to evaluate TNFα production by immune and non-immune decidua cells as denoted by MFI. Low baseline TNFα production by DMs in Controls did not change after LPS-exposure (**Figure 6B**). Similarly, T cell and CD45 negative decidua cell TNFα production also was low with no further increase after LPS-exposure. However, TNFα production increased in decidua neutrophils after LPS-exposure denoted by a clear shift in MFI, an effect that was attenuated by Adalimumab treatment (**Figure 6B**). In contrast, DM TNFα production was much more modest with no significant increase in LPS-exposed vs Control DMs (**Figure 6C**).

To validate these findings, we conducted identical experiments in human samples. Intracellular TNFα production was evaluated in freshly dissociated human decidua cells collected soon after delivery without exogenous stimulation. Both human neutrophils and DMs showed a low-baseline TNFα production (**Figure 6D**). Similar to our observations in the Rhesus model, *ex vivo* human DMs, T cells, CD45 negative cells demonstrated no significant differences in TNFα production between Chorio - and Chorio + groups (**Figure 6D**). In contrast to Rhesus, chorioamnionitis did not increase TNFα production in human decidua neutrophils (**Figure 6D**). The differences between Rhesus macaque and human decidua neutrophil TNFα production may be due to a more sub-acute rather than acute inflammatory process in the humans. Alternatively, human pathology may be due to lower virulence micro-organisms causing chorioamnionitis but minimally increasing TNFα production e.g. Ureaplasma species (21). Similar to the Rhesus, human DM TNFα production was also modest with no significant increase in Chorio+ vs Chorio- DMs (**Figure 6E**).

To assess the impact of *in utero* exposure on DM responsiveness to a secondary inflammatory challenge, intracellular TNFα was measured in thawed, previously cryopreserved DMs following *in vitro* LPS stimulation (**Figure 6F**). As with the *ex vivo* assays, total dissociated decidua was utilized and cultured overnight without stimulation to allow recovery after thawing. At baseline, Rhesus DMs lacked detectable TNFα expression across all groups (**Figure 6G**). Upon *in vitro* LPS challenge, Rhesus DMs from Control and Adal+LPS groups exhibited a significant increase in TNFα MFI, whereas the frequency of TNFα^+^ DMs did not significantly change in any group (**Figure 6G**). Notably, the *in vivo* LPS-exposed group failed to respond to the secondary *in vitro* LPS challenge both in MFI and frequency. In the human DMs, a low baseline TNFα production was noted in both Chorio - and Chorio + donors (**Figure 6H**). Surprisingly, after *in vitro* LPS challenge, human DMs regardless of Chorio exposure failed to upregulate TNFα production (**Figure 6H**). These results of non-responsiveness to a secondary LPS challenge could be due to “endotoxin tolerance” in which a prior exposure to LPS programs cells to be refractory to a subsequent LPS challenge (22), or to upregulation of negative regulators of the inflammatory pathway. We did not observe differential expression of classic endotoxin tolerance-associated genes, such as *IRAK3* and *SOCS1* in our transcriptomic data (**Supplementary Figure S12**). However, several negative regulators of NF-kB pathway were upregulated after LPS exposure including *NFKBIA, NFKBIZ, TNFAPI3*, and *ATF3* (**Supplementary Figure S12**), suggesting that the lack of *in vitro* LPS responsiveness with increase in TNFα production may be driven by negative regulatory pathway induced in DMs rather than the canonical endotoxin tolerance pathway activation. A suppressive decidua milieu may also blunt LPS responsiveness of decidua DMs.

### 2.9 Decidua macrophages of fetal origin can mount an inflammatory response

**Figure 7:**
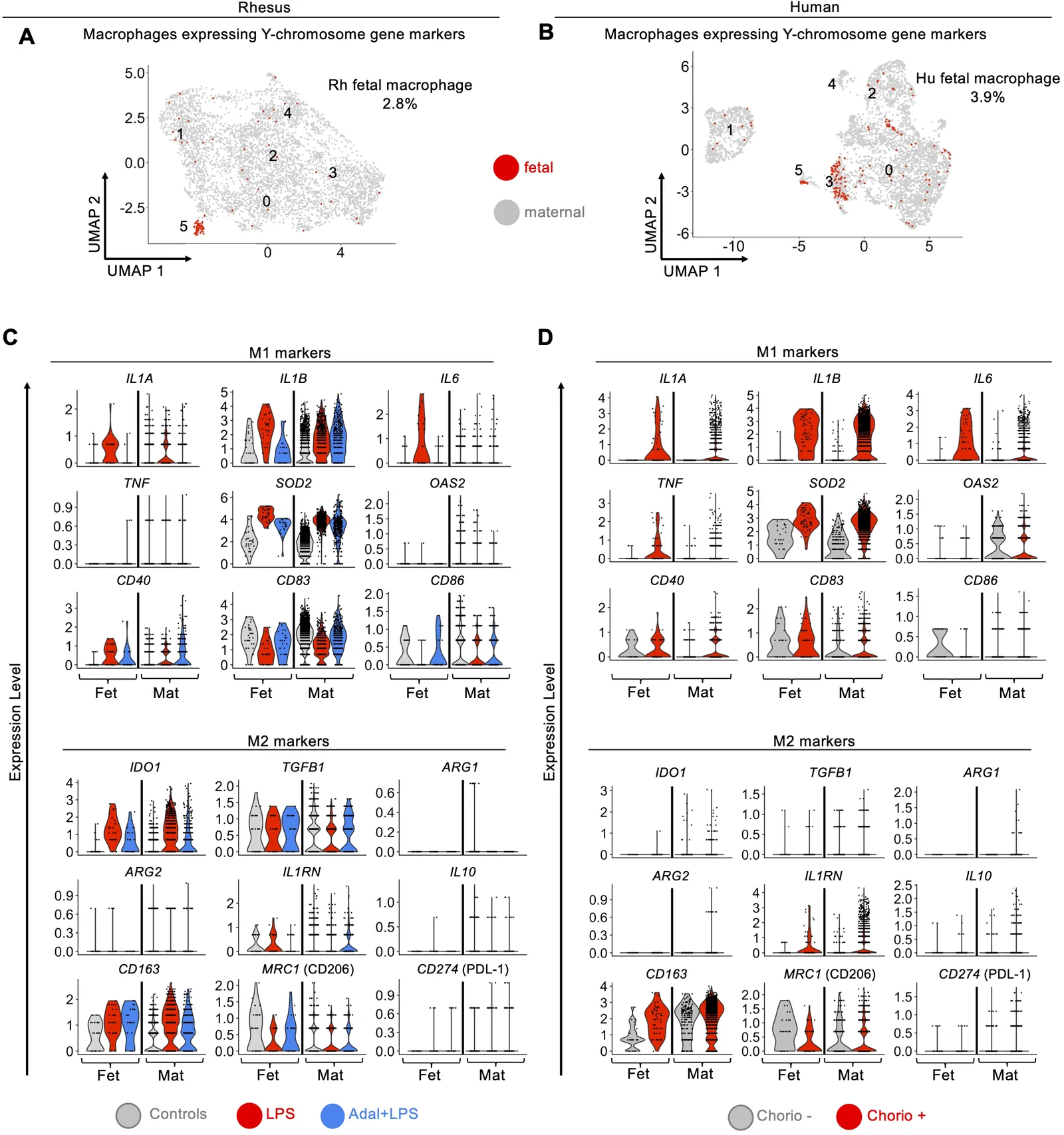
Fetal DMs participate in the inflammatory response during chorioamnionitis. **(A)** Rhesus DM cluster 5 and **(B)** human DM clusters 3 and 5 predominantly expresses Y-chromosome markers confirming their fetal origin. **(C, D)** Violin plots of selected pro- and anti-inflammatory gene markers show that fetal origin DMs showed similar or increased expression of several markers compared to their maternal counterparts.

DM cluster 5 in the Rhesus and clusters 3, 5 in human contained cells expressing Y-chromosome genes (**Figures 7A, B**), indicating male fetus origin. These male fetus DMs accounted for 89/3109 DMs (2.8%) in the Rhesus and 83/2126 DMs (3.9%) in human. Although fetal macrophages were a small fraction of the total DM population, the distinct UMAP space occupied by these cells suggested that they are transcriptionally distinct from maternal cells. Next, we compared responses to intrauterine inflammation by separately analyzing the scRNAseq datasets of male fetal vs. maternal DMs (**Figures 7C, D**). In LPS-exposed Rhesus macaques, fetal DMs upregulated 51 DEGs compared to maternal DMs, whereas human fetal DMs upregulated 47 DEGs compared to maternal DMs in chorioamnionitis-exposed subjects (FDR adjusted p value ≤ 0.05, log2FC 1, **Supplementary Table 12**). Functional annotation analyses of these DEGs revealed a significant enrichment of Gene Ontology (GO) terms associated with inflammation-related processes including “inflammatory response” and “regulation of cell proliferation” in both species (**Supplementary Figures S13A, B**; **Supplementary Table 12**). In contrast to upregulated genes, fetal downregulated genes were fewer in number. In LPS-exposed Rhesus macaques, fetal DMs downregulated 2 DEGs (*MGST3*, *PYCARD*) compared to maternal DMs, whereas human fetal DMs downregulated 9 DEGs compared to maternal DMs in chorioamnionitis-exposed subjects (*CD74*, *SRGN*, *PASP*, *LYZ*, *IFI30*, *APOE*, *TMSB10*, *FOS*, *CTSS*) (FDR adjusted p value ≤ 0.05, log2FC 1; **Supplementary Table 12**).

A comparison of Rhesus vs human fetal origin DMs revealed largely overlapping expression patterns: expression levels for cytokines *IL1A, IL1B*, and *SOD2* appeared to be the same or higher in fetal compared to maternal DMs during chorioamnionitis. Crucially, the upregulation of *IL6* during chorioamnionitis was almost exclusively restricted to fetal but not maternal DMs (**Figures 7C, D**). Species-specific variations were also noted: the M2 markers *IDO1* and *TGFB1* were expressed in Rhesus fetal and maternal DMs but were absent in human DMs. Furthermore, *TNF* was not expressed in either Rhesus fetal or maternal DMs, whereas fetal macrophages appeared to increase *TNF* expression in human chorioamnionitis. In both species, chorioamnionitis exposure increased *CD163*, decreased *MRC1* (CD206) expression, while *CD274* (PDL1) and *ARG2* were minimally expressed in fetal and maternal DMs.

Collectively, these data demonstrate that although fetal origin DMs are rare (≈3–4%), they selectively upregulate *IL6* and inflammation-associated pathways during chorioamnionitis, raising the possibility of a localized fetal contribution to decidual immunity.

## 3 Discussion

Maternal host defense during chorioamnionitis poses an existential challenge of balancing anti-microbial immune response with fetal tolerance. In this pre-clinical study, we show that many of the anti-inflammatory genes (e.g. *IL1RN, TNIP3, IDO1/2*) are coordinately upregulated in the decidua macrophages (DMs) along with pro-inflammatory genes during chorioamnionitis. DMs exhibit a hybrid M1/M2 polarization that do not significantly change during chorioamnionitis. A noteworthy rigor of this study is that the findings were corroborated by multiple assays including bulk RNAseq of flow sorted DMs, scRNAseq, and flow cytometry in both human and Rhesus macaque. Overall, our data demonstrate that decidual macrophages (DMs) at the maternal–fetal interface mount a restrained rather than a pro-inflammatory response to acute chorioamnionitis. Notably, TNFα production was low at baseline and during chorioamnionitis. Yet paradoxically TNF signaling inhibition suppressed ~50% of LPS-induced genes in DMs.

Our results show that Rhesus macaque decidua neutrophils as the primary source of TNFα in LPS-exposed DMs, an effect that was attenuated by TNF-blockade. Thus, TNF-blockade appears to be acting on the DMs indirectly via effects on neutrophils. This paracrine axis may underscore TNF-blockade as a viable therapeutic strategy to dampen intrauterine inflammation without completely disrupting the baseline homeostatic functions of tissue-resident DMs.

An important finding both in RNAseq and flow cytometry data was that in response to LPS DMs increased expression of *CCL2* and *IFNB1* but not *TNF, IL6, IL12, CXCL10*. Furthermore, we found that after an *in vitro* LPS challenge DMs from human donors did not produce TNFα, while Rhesus DMs only from control animals responded with increased TNFα expression. Interestingly, DMs from *in vivo* LPS exposed Rhesus macaques were refractory to an *in vitro* LPS challenge likely due to upregulation of negative regulators of NF-kB signaling. Our results of lack of TNFα production by DMs during chorioamnionitis are at odds with prior publications showing TNFα production by DMs during spontaneous preterm labor (4, 23), and DMs producing TNFα *in vitro* in response to LPS (24). A possible explanation for the discrepancy is different study designs in these experiments. In our study, DMs mixed with other decidua cells were used for challenge with LPS to simulate *in vivo* conditions. On the other hand, previous studies used DMs purified by FACS or plate adhesion for LPS challenge (24). Our results suggesting possible inhibition of DM TNF production by decidua milieu are consistent previous studies demonstrating extra-villous trophoblasts and decidua stroma cells suppressing macrophage TNFα and IL12 production (25, 26). Of note, DMs from control Rhesus macaques were capable of TNFα production, while human DMs were refractory to a LPS challenge. Previous clinical studies reported that DMs can express TNFα (4, 23). However, we report no/minimal detection of *in vivo* TNFα production in the present study. The discrepancies could be due to differences in patient populations, different micro-organisms e.g. low-virulence *Ureaplasma* spp. vs virulent gram-negative bacteria, or different clinical diagnoses.

Despite the lack of upregulation of several pro-inflammatory cytokines including TNF in both human and Rhesus macaque DMs during chorioamnionitis, upstream regulator and pathway analysis of bulk RNAseq of Rhesus DMs revealed that *TNF, IL1, IFNG, STAT3, IL17A* signaling was upregulated by LPS. TNF signaling pathway was active in the DM, since inhibition of TNF downregulated ~50% of the DM genes induced by LPS. If macrophages did not produce TNF but responded to TNF signals during chorioamnionitis, which are the TNF producing cells? We previously reported that recruited neutrophils during chorioamnionitis are the major producers of TNFα, myeloperoxidase, and PTGS2 (6, 27).

IL6, another highly upregulated cytokine during chorioamnionitis, is expressed mainly by the amniotic mesenchymal cells and the decidua stroma cells (27, 28). Of note, our data demonstrating upregulation of *CSF3* expression during chorioamnionitis predicts DMs playing a role in neutrophil recruitment and function indicating a delicately balanced paracrine signaling loop between DMs, neutrophils and other decidua cells. Collectively, our data suggest that DMs coordinate innate immune responses at the maternal–fetal interface without themselves substantially amplifying pro-inflammatory cytokine production during chorioamnionitis.

Inflammation is known to play a major role in initiating labor (29). Earlier studies reported that upregulation of pro-inflammatory cytokines by DMs initiate both term and preterm labor (30–32). However, a more recent study suggested that DMs are homeostatic and are required to sustain pregnancy and prevent preterm births (4). In our study, Rhesus macaques did not have preterm labor and 3/10 human subjects had spontaneous preterm labor. Thus, our study was not designed to definitively address the role of DM in preterm labor/birth. However, our findings that classic M1 pro-inflammatory cytokines were not upregulated by LPS in the rhesus DMs and TNF expression was not detectable during chorioamnionitis in human DMs are consistent with a homeostatic role for DM during pregnancy.

Macrophage responses to external stimuli have been understood to be broadly categorized as M1 or M2 phenotype (8). These M1/M2 functional states are associated with different sets of transcription factors, cytokines and cell surface markers subserving different functions during health and disease states (18). For e.g. M1 macrophages utilize TLR/NF-kB p50p65 to induce genes for *TNF, IL12, CCL2*, nitric oxide production, and *IFN/IRF3/5* axis regulating *CXCL9/10/11* genes. These inflammatory changes help with bacterial and viral host defense through recruitment of neutrophils and activating the Th1 arm of T cells (33). On the other hand, M2 macrophages utilize IL4/IL13 Stat6 axis to induce genes for *SOCS1, ARG1, PPAR*, stimulating activity of mast cells, basophils, and B cells, and Th2 arm of T cells (33). M2 macrophages in concert with other immune cells play an important role in allergic diseases, parasitic infections and wound healing.

Previous studies had suggested that during pregnancy DMs transition from M1 state during early pregnancy, with predominant M2-like phenotype for rest of the pregnancy until parturition when M1 predominance returns (23, 34, 35). However, other studies also found a mixed M1 and M2 phenotype and function of two DM populations characterized by CD11c^LO^ and CD11c^HI^ expression (24). These changes in macrophage phenotypes throughout pregnancy should be viewed in context with other immune cells in the decidua. In the first trimester, NK cells are the most abundant immune cells guiding trophoblast invasion and remodeling of spiral artery important for placentation (36). T regulatory cells are critical for creating a tolerogenic milieu for the fetus antigenically distinct from the mother (37, 38). During progression towards parturition in the third trimester, decidua adopts a more pro-inflammatory state. In this study, we solidify that M1 and M2 functional markers are co-expressed at least in some DM types suggesting a true hybrid state. This observation is distinct from previously described plasticity of macrophages, wherein macrophages transitioned from one state to another during different phases of disease e.g. M1 during acute inflammation transitioning to M2 during tissue repair. Notably, the findings were confirmed both in human and Rhesus macaque third trimester DMs. This is significant because human DMs from preterm pregnancy could be influenced by the underlying condition causing prematurity, while Rhesus macaques in the control group were healthy with a normal pregnancy history. Overall, our data strongly suggest that third trimester DMs exhibit a hybrid M1/M2 polarity that do not change during chorioamnionitis.

A novel finding in our study is that DMs of fetal origin comprise 3-4% of the total DM population and they are transcriptionally active but distinct from maternal DMs based on their distinct UMAP distribution. Furthermore, both Rhesus and human fetal origin DMs selectively upregulate *IL6* during chorioamnionitis, raising the possibility of a fetal contribution to decidual immunity; however, their overall functional impact relative to maternal DMs remains to be determined. A prior study of first trimester placenta had identified antimicrobial efficacy of Hoffbauer cells which are macrophages in placental villi (39). Collectively, these data suggest that fetal origin DMs both in the placental villi and in the decidua may play an active participant in the host immune response to inflammation or infection in the decidua.

A few limitations of the study are worth noting. First, microbial identity causing chorioamnionitis in human subjects was not available. Most common organisms identified during chorioamnionitis in the western world are the *Ureaplasma* species (40). Second, our Rhesus macaque LPS-exposure was short (16h) and the exposure period to chorioamnionitis in human subjects was not known. DM responses to inflammation may be different during acute vs. chronic stages. Third, our human scRNAseq datasets were derived from formalin-fixed paraffin-embedded (FFPE) blocks. This processing approach can introduce significant cellular bias, as fragile effector populations, particularly neutrophils, may not be adequately represented. Finally, our clinical validation cohort remains small (n=10 total, with n=5 confirmed chorioamnionitis cases), with scRNAseq data derived from two cases per group. Given the extensive clinical heterogeneity of chorioamnionitis including variations in microbial etiology, gestational age, duration of rupture, and antenatal corticosteroid exposure, larger, prospective multi-center validation cohorts are required to confirm these regulatory paradigms.

In conclusion, macrophages from decidua parietalis in the third trimester of pregnancy express both M1 and M2 markers that do not significantly change during chorioamnionitis. Both pro- and anti-inflammatory cytokines are upregulated in the DMs during chorioamnionitis. Although DMs do not express *TNF* during chorioamnionitis, TNF signaling plays an important role in orchestrating DM immune response that carefully balances host defense with a restrained pro-inflammatory profile to potentially prevent adverse outcomes from chorioamnionitis. Activation of fetal origin macrophages during chorioamnionitis may have implications in shaping neonatal immune responses.

## 4 Methods

Detailed methods are in the supplement.

### 4.1 Animal samples

Normal cycling, adult female Rhesus macaques (*Macaca mulatta*) (n=33) were time mated. Pregnant rhesus received either 1mL saline (n=11) or 1mg LPS (Sigma-Aldrich, St. Louis, MO, n=12) in a 1mL saline solution. Saline and LPS were delivered via ultrasound guided intraamniotic (IA) injection at ~130 days of gestation (~80% term gestation). Adalimumab (Humira, AbbVie Inc. North Chicago, IL), a Tumor Necrosis Factor (TNF) blocker, was used in order to block TNF signaling. Adalimumab (n=10) was given IA (40mg) + maternal subcutaneous (SC) (40mg) 1 and 3 hours before LPS, respectively) (**Supplementary Figure S1A**). Surgical delivery of the fetus occurred 16 hours after LPS-exposure. This was determined to be the optimum timing based off previous studies (6, 7). Maternal and neonatal demographic characteristics are shown in **Supplementary Table 1A**. Preterm labor and spontaneous death did not occur in any animals. For some experiments, animals from older studies were also used in order to increase sample size. The numbers of samples for each experiment are shown in **Supplementary Table 1B**.

### 4.2 Human samples

Eleven pregnant women with preterm pregnancies (26^0^ to 35^6^ weeks of gestation) had been recruited. Clinical demographic characteristics and numbers of sample donors for different experiments are shown in **Supplementary Tables 6A, B**). Placental pathology was used to determine histologic chorioamnionitis (Chorio) based on Redline’s criteria (41). Cohorts were developed based on Chorio positive (+) classification (Chorio - n=6 and Chorio + n=5). Various preterm samples were used in prior studies (6, 10).

### 4.3 Chorioamnion-decidua cell isolation and bulk RNAseq

Rhesus and human extra-placental chorioamnion-decidua fetal membranes were collected within 30 min of delivery. Tissue was dissected away from the placenta and cell suspensions were used for different experiments as reported (7, 42). Bulk RNAseq was done using procedures reported previously (7). Cell-type signature analysis was performed using SaVanT (43). Custom macrophage signatures for M1 and M2 were generated by integrating gene sets from **Supplementary Table 3** and uploaded using SaVanT’s custom signature option. Raw gene-level count matrices for each sample were provided as input. SaVanT was run in sample-level mode to compute signature scores for all samples using the custom macrophage reference.

### 4.4 Single-cell (sc)RNAseq: Library construction, sequencing and analysis

Fresh chorioamnion-decidua tissue in the Rhesus and formalin fixed paraffin embedded tissue in the human were used for scRNAseq. Library construction, sequencing, quality control and analysis were done as described (9, 10). Canonical markers were used to annotate the cell types based on marker genes. Gene signature scoring was performed using the UCell package (AddModuleScore_UCell) to calculate module scores at single-cell resolution, and smoothed signature distributions were computed using SmoothKNN on Harmony embeddings.

### 4.5 Flow cytometry

Monoclonal antibodies (mAbs) used for FACS (**Figure 1A**), immunophenotype (**Supplementary Figure S2**), functional studies (**Figure 6A**), and M1/M2 characterization (**Figure 7**) are listed in **Supplementary Tables 11A, B**. All antibodies were titrated for optimal detection of positive populations and mean fluorescence intensity (MFI). Immunophenotyping and functional studies were done as described (6, 7, 42) Rhesus decidua macrophages were defined as CD45^+^CD3^-^CD19^-^CD20^-^CD56^-^CD14^high^CD88^+^HLA-DR^+^ cells. Intracellular TNFα was assessed: 1) *in vitro*: decidua cells were thawed and cultured overnight allowing them to rest and maximize cytokine production. Cells were then stimulated with LPS (Sigma-Aldrich, St. Louis, MO) for 3 hours. Golgi Stop (BD Bioscience) was added for the last two hours; 2) *ex vivo*: after isolation, decidua cells were immediately stained for flow cytometry analysis without any stimulation and/or intracellular blockers, as previously described (6). Data were analyzed using FlowJo version 10.10.0 software (TreeStar Inc.).

### 4.6 Statistical analysis

Statistical significance was analyzed and graphed using GraphPad Prism software (version 9.0). Values were expressed as means ± SEM. Fisher’s exact test (for categorical variables), two-tailed Mann-Whitney U tests (for non-normally distributed continuous variables), 2-tailed Student t test (for Gaussian distributed data points), and two-way ANOVA test were used to determine differences between groups. Results were considered significant for p ≤ 0.05.

## Data Availability

The datasets presented in this study can be found in online repositories. The names of the repository/repositories and accession numbers can be found below: https://www.ncbi.nlm.nih.gov/geo/, GSE243830 https://www.ncbi.nlm.nih.gov/geo/, GSE300221 https://www.ncbi.nlm.nih.gov/geo/, GSE282837.

## References

[1] Gosselin D Link VM Romanoski CE Fonseca GJ Eichenfield DZ Spann NJ . Environment drives selection and function of enhancers controlling tissue-specific macrophage identities. Cell. (2014) 159:1327–40. doi: 10.1016/j.cell.2014.11.023 25480297 PMC4364385

[2] Cappelletti M Presicce P Kallapur SG . Immunobiology of acute chorioamnionitis. Front Immunol. (2020) 11:649. doi: 10.3389/fimmu.2020.00649 32373122 PMC7177011

[3] Jiang X Du MR Li M Wang H . Three macrophage subsets are identified in the uterus during early human pregnancy. Cell Mol Immunol. (2018) 15:1027–37. doi: 10.1038/s41423-018-0008-0 29618777 PMC6269440

[4] Gomez-Lopez N Garcia-Flores V Chin PY Groome HM Bijland MT Diener KR . Macrophages exert homeostatic actions in pregnancy to protect against preterm birth and fetal inflammatory injury. JCI Insight. (2021) 6. doi: 10.1172/jci.insight.146089 34622802 PMC8525593

[5] Wheeler KC Jena MK Pradhan BS Nayak N Das S Hsu CD . Vegf may contribute to macrophage recruitment and m2 polarization in the decidua. PloS One. (2018) 13:e0191040. doi: 10.1371/journal.pone.0191040 29324807 PMC5764356

[6] Presicce P Park CW Senthamaraikannan P Bhattacharyya S Jackson C Kong F . Il-1 signaling mediates intrauterine inflammation and chorio-decidua neutrophil recruitment and activation. JCI Insight. (2018) 3. doi: 10.1172/jci.insight.98306 29563340 PMC5926925

[7] Presicce P Cappelletti M Senthamaraikannan P Ma F Morselli M Jackson CM . Tnf-signaling modulates neutrophil-mediated immunity at the feto-maternal interface during lps-induced intrauterine inflammation. Front Immunol. (2020) 11:558. doi: 10.3389/fimmu.2020.00558 32308656 PMC7145904

[8] Mills CD Kincaid K Alt JM Heilman MJ Hill AM . M-1/M-2 macrophages and the th1/th2 paradigm. J Immunol. (2000) 164:6166–73. doi: 10.4049/jimmunol.164.12.6166 10843666

[9] Presicce P Cappelletti M Morselli M Ma F Senthamaraikannan P Protti G . Amnion responses to intrauterine inflammation and effects of inhibition of tnf signaling in preterm rhesus macaque. iScience. (2023) 26:108118. doi: 10.1016/j.isci.2023.108118 37953944 PMC10637919

[10] Prasanphanich NS Protti G Cappelletti M Ma F Pithia N Dasenbrock A . Granulysin-high decidual nk cells in macaques and humans share signatures of immune defense. Cell Rep. (2025) 44:115943. doi: 10.1016/j.celrep.2025.115943 40638390 PMC12329683

[11] Suryawanshi H Morozov P Straus A Sahasrabudhe N Max KEA Garzia A . A single-cell survey of the human first-trimester placenta and decidua. Sci Adv. (2018) 4:eaau4788. doi: 10.1126/sciadv.aau4788 30402542 PMC6209386

[12] Vento-Tormo R Efremova M Botting RA Turco MY Vento-Tormo M Meyer KB . Single-cell reconstruction of the early maternal-fetal interface in humans. Nature. (2018) 563:347–53. doi: 10.1038/s41586-018-0698-6 30429548 PMC7612850

[13] Pique-Regi R Romero R Tarca AL Sendler ED Xu Y Garcia-Flores V . Single cell transcriptional signatures of the human placenta in term and preterm parturition. Elife. (2019) 8. doi: 10.7554/eLife.52004 31829938 PMC6949028

[14] Nishikoba N Kumagai K Kanmura S Nakamura Y Ono M Eguchi H . Hgf-met signaling shifts m1 macrophages toward an m2-like phenotype through pi3k-mediated induction of arginase-1 expression. Front Immunol. (2020) 11:2135. doi: 10.3389/fimmu.2020.02135 32983173 PMC7492554

[15] Villani AC Satija R Reynolds G Sarkizova S Shekhar K Fletcher J . Single-cell rna-seq reveals new types of human blood dendritic cells, monocytes, and progenitors. Science. (2017) 356. doi: 10.1126/science.aah4573 28428369 PMC5775029

[16] Liu MJ Bao S Galvez-Peralta M Pyle CJ Rudawsky AC Pavlovicz RE . Zip8 regulates host defense through zinc-mediated inhibition of nf-kappab. Cell Rep. (2013) 3:386–400. doi: 10.1016/j.celrep.2013.01.009 23403290 PMC3615478

[17] Pusztai C Yusenko MV Banyai D Szanto A Kovacs G . M2 macrophage marker chitinase 3-like 2 (chi3l2) associates with progression of conventional renal cell carcinoma. Anticancer Res. (2019) 39:6939–43. doi: 10.21873/anticanres.13915 31810965

[18] Sica A Mantovani A . Macrophage plasticity and polarization: *In vivo* veritas. J Clin Invest. (2012) 122:787–95. doi: 10.1172/JCI59643 22378047 PMC3287223

[19] Shapouri-Moghaddam A Mohammadian S Vazini H Taghadosi M Esmaeili SA Mardani F . Macrophage plasticity, polarization, and function in health and disease. J Cell Physiol. (2018) 233:6425–40. doi: 10.1002/jcp.26429 29319160 PMC13482560

[20] Jang SH Choi H Lee EM Yoon SB Kim HJ Park C . Single-cell analysis of the decidua unveils the mechanism of anti-inflammatory exosomes for chorioamnionitis in nonhuman primates. Sci Adv. (2025) 11:eadp0467. doi: 10.1126/sciadv.adp0467 40601753 PMC12219548

[21] Senthamaraikannan P Presicce P Rueda CM Maneenil G Schmidt AF Miller LA . Intra-amniotic ureaplasma parvum-induced maternal and fetal inflammation and immune responses in rhesus macaques. J Infect Dis. (2016) 214:1597–604. doi: 10.1093/infdis/jiw408 27601620 PMC6392471

[22] Kallapur SG Jobe AH Ball MK Nitsos I Moss TJ Hillman NH . Pulmonary and systemic endotoxin tolerance in preterm fetal sheep exposed to chorioamnionitis. J Immunol. (2007) 179:8491–9. doi: 10.4049/jimmunol.179.12.8491 18056396

[23] Xu Y Romero R Miller D Kadam L Mial TN Plazyo O . An m1-like macrophage polarization in decidual tissue during spontaneous preterm labor that is attenuated by rosiglitazone treatment. J Immunol. (2016) 196:2476–91. doi: 10.4049/jimmunol.1502055 26889045 PMC4779725

[24] Houser BL Tilburgs T Hill J Nicotra ML Strominger JL . Two unique human decidual macrophage populations. J Immunol. (2011) 186:2633–42. doi: 10.4049/jimmunol.1003153 21257965 PMC3712354

[25] Eastman AJ Vrana EN Grimaldo MT Jones AD Rogers LM Alcendor DJ . Cytotrophoblasts suppress macrophage-mediated inflammation through a contact-dependent mechanism. Am J Reprod Immunol. (2021) 85:e13352. doi: 10.1111/aji.13352 32969101 PMC7897285

[26] Svensson-Arvelund J Mehta RB Lindau R Mirrasekhian E Rodriguez-Martinez H Berg G . The human fetal placenta promotes tolerance against the semiallogeneic fetus by inducing regulatory t cells and homeostatic m2 macrophages. J Immunol. (2015) 194:1534–44. doi: 10.4049/jimmunol.1401536 25560409

[27] Cappelletti M Presicce P Ma F Senthamaraikannan P Miller LA Pellegrini M . The induction of preterm labor in rhesus macaques is determined by the strength of immune response to intrauterine infection. PloS Biol. (2021) 19:e3001385. doi: 10.1371/journal.pbio.3001385 34495952 PMC8452070

[28] Presicce P Roland C Senthamaraikannan P Cappelletti M Hammons M Miller LA . Il-1 and tnf mediates il-6 signaling at the maternal-fetal interface during intrauterine inflammation. Front Immunol. (2024) 15:1416162. doi: 10.3389/fimmu.2024.1416162 38895127 PMC11183269

[29] Leimert KB Xu W Princ MM Chemtob S Olson DM . Inflammatory amplification: A central tenet of uterine transition for labor. Front Cell Infect Microbiol. (2021) 11:660983. doi: 10.3389/fcimb.2021.660983 34490133 PMC8417473

[30] Osman I Young A Ledingham MA Thomson AJ Jordan F Greer IA . Leukocyte density and pro-inflammatory cytokine expression in human fetal membranes, decidua, cervix and myometrium before and during labour at term. Mol Hum Reprod. (2003) 9:41–5. doi: 10.1093/molehr/gag001 12529419

[31] Hamilton S Oomomian Y Stephen G Shynlova O Tower CL Garrod A . Macrophages infiltrate the human and rat decidua during term and preterm labor: Evidence that decidual inflammation precedes labor. Biol Reprod. (2012) 86:39. doi: 10.1095/biolreprod.111.095505 22011391

[32] Shynlova O Nedd-Roderique T Li Y Dorogin A Nguyen T Lye SJ . Infiltration of myeloid cells into decidua is a critical early event in the labour cascade and post-partum uterine remodelling. J Cell Mol Med. (2013) 17:311–24. doi: 10.1111/jcmm.12012 23379349 PMC3822594

[33] Mills CD . Anatomy of a discovery: M1 and M2 macrophages. Front Immunol. (2015) 6:212. doi: 10.3389/fimmu.2015.00212 25999950 PMC4419847

[34] Brown MB von Chamier M Allam AB Reyes L . M1/M2 macrophage polarity in normal and complicated pregnancy. Front Immunol. (2014) 5:606. doi: 10.3389/fimmu.2014.00606 25505471 PMC4241843

[35] Svensson J Jenmalm MC Matussek A Geffers R Berg G Ernerudh J . Macrophages at the fetal-maternal interface express markers of alternative activation and are induced by m-csf and il-10. J Immunol. (2011) 187:3671–82. doi: 10.4049/jimmunol.1100130 21890660

[36] Croy BA Chantakru S Esadeg S Ashkar AA Wei Q . Decidual natural killer cells: Key regulators of placental development (a review). J Reprod Immunol. (2002) 57:151–68. doi: 10.1016/s0165-0378(02)00005-0 12385840

[37] Gomez-Lopez N Galaz J Miller D Farias-Jofre M Liu Z Arenas-Hernandez M . The immunobiology of preterm labor and birth: Intra-amniotic inflammation or breakdown of maternal-fetal homeostasis. Reproduction. (2022) 164:R11–45. doi: 10.1530/REP-22-0046 35559791 PMC9233101

[38] Rowe JH Ertelt JM Aguilera MN Farrar MA Way SS . Foxp3(+) regulatory t cell expansion required for sustaining pregnancy compromises host defense against prenatal bacterial pathogens. Cell Host Microbe. (2011) 10:54–64. doi: 10.1016/j.chom.2011.06.005 21767812 PMC3140139

[39] Thomas JR Appios A Zhao X Dutkiewicz R Donde M Lee CYC . Phenotypic and functional characterization of first-trimester human placental macrophages, hofbauer cells. J Exp Med. (2021) 218. doi: 10.1084/jem.20200891 33075123 PMC7579740

[40] Romero R Pacora P Kusanovic JP Jung E Panaitescu B Maymon E . Clinical chorioamnionitis at term x: Microbiology, clinical signs, placental pathology, and neonatal bacteremia - implications for clinical care. J Perinatal Med. (2021) 49:275–98. doi: 10.1515/jpm-2020-0297 33544519 PMC8324070

[41] Redline RW Faye-Petersen O Heller D Qureshi F Savell V Vogler C . Amniotic infection syndrome: Nosology and reproducibility of placental reaction patterns. Pediatr Dev Pathol. (2003) 6:435–48. doi: 10.1007/s10024-003-7070-y 14708737

[42] Presicce P Senthamaraikannan P Alvarez M Rueda CM Cappelletti M Miller LA . Neutrophil recruitment and activation in decidua with intra-amniotic il-1beta in the preterm rhesus macaque. Biol Reprod. (2015) 92:56. doi: 10.1095/biolreprod.114.124420 25537373 PMC4342792

[43] Lopez D Montoya D Ambrose M Lam L Briscoe L Adams C . Savant: A web-based tool for the sample-level visualization of molecular signatures in gene expression profiles. BMC Genomics. (2017) 18:824. doi: 10.1186/s12864-017-4167-7 29070035 PMC5657101

