# Supplementary material for: Decidua macrophages display a restrained inflammatory profile during chorioamnionitis
Source: Front Immunol. 2026 Aug 10;17:1858982. doi: 10.3389/fimmu.2026.1858982 (PMC13500772; doi:10.3389/fimmu.2026.1858982)
Supplement: Supplementary file 1 [file DataSheet1.pdf]

Fig. S1

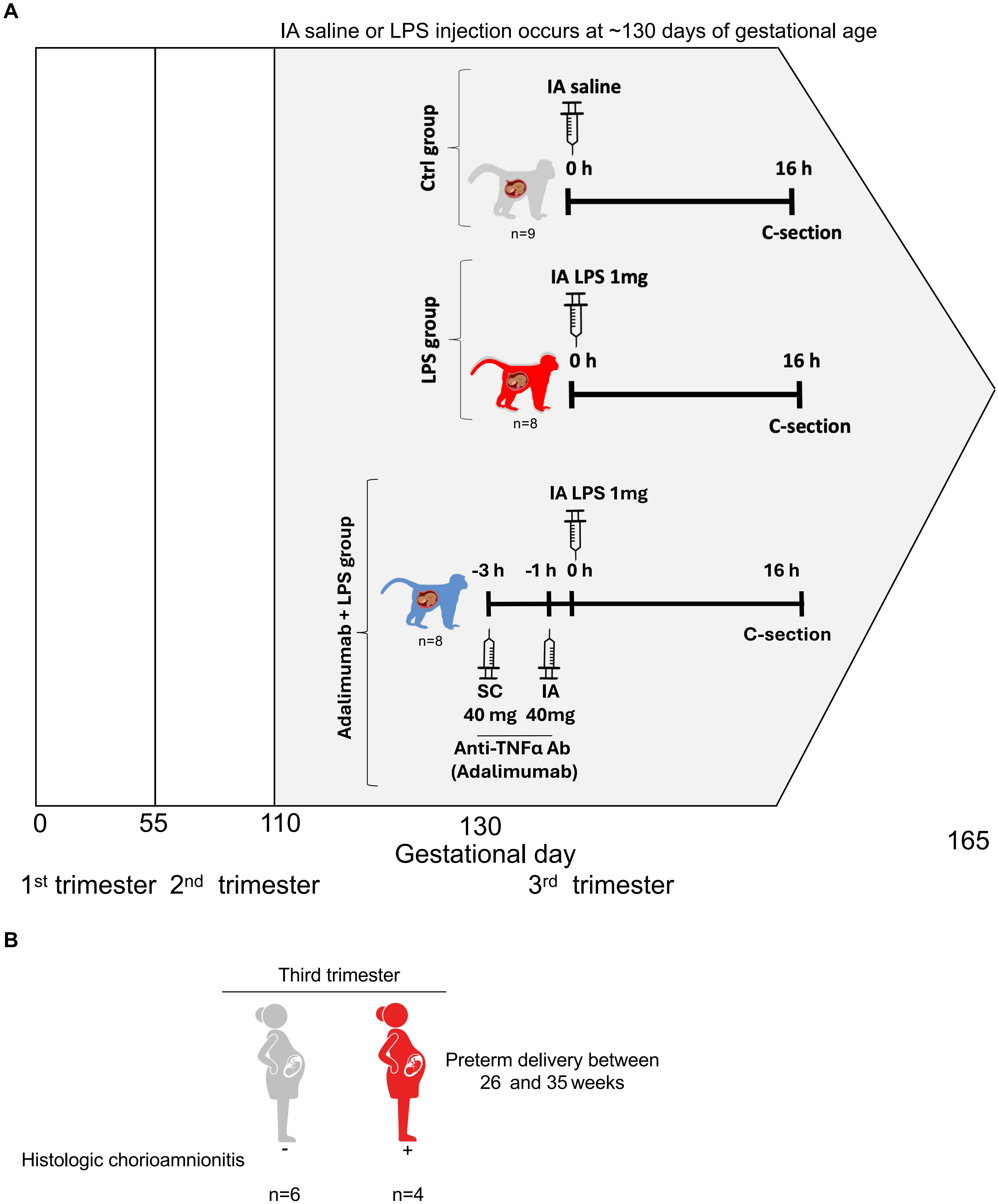

Fig. S1. Experimental design of used in the study. **A** Three groups of pregnant Rhesus macaques at approximately 80% gestation were administered intraamniotic (IA) injections of either lipopolysaccharide (LPS) or saline. One of the LPS-treated groups received the TNF-α inhibitor Adalimumab via subcutaneous (SC) and IA injection prior to IA LPS administration. **B** Human samples were collected from women who delivered preterm, with or without histologic evidence of chorioamnionitis.

Fig. S2

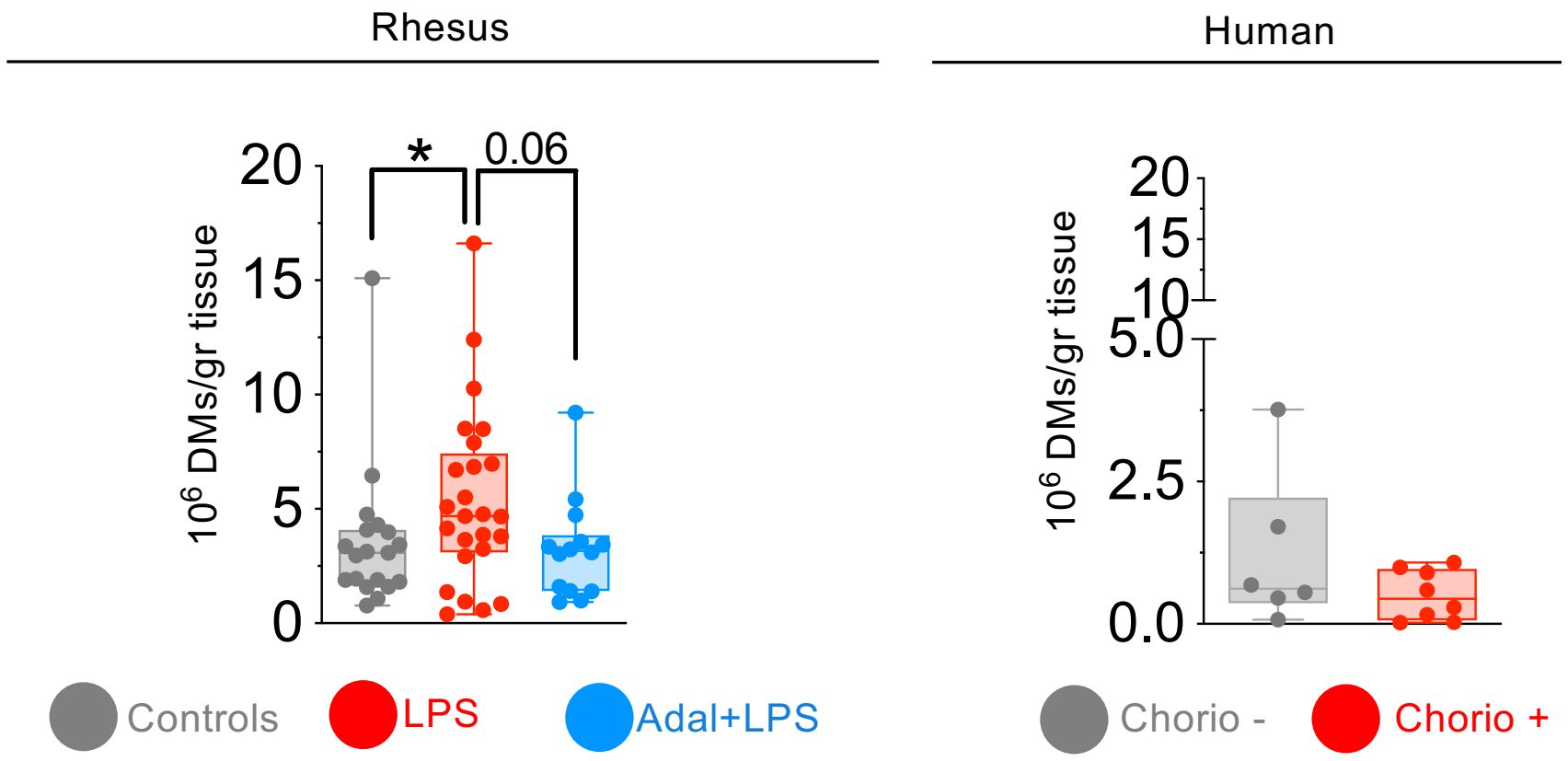

Fig. S2. Chorioamnionitis increased the number of DMs in Rhesus but not in human. Cell suspensions from Rhesus and human decidua parietalis were analyzed by multi-parameter flow cytometry. Rhesus DMs were defined as Live cells/$CD45^{+}CD3^{-}CD19^{-}CD20^{-}CD56^{-}CD14^{high}CD88^{+}HLA\text{-}DR^{+}$ cells while human DMs were defined as Live cells/$CD45^{+}CD3^{-}CD19^{-}CD20^{-}CD56^{-}CD66b^{-}CD14^{high}HLA\text{-}DR^{+}$ cells. In Rhesus, LPS exposure increased significantly the number of DMs and TNF-inhibition by Adalimumab decreased it although not significantly (Controls n=19; LPS n=25; Adal+LPS n=14). In human, no difference in DM numbers were observed between Chorio – and Chorio + samples (Chorio – n=6; Chorio + n=8). (Data are mean, SEM, *$p<0.05$ between comparators by Mann-Whitney test). Some of samples used in these graphs were also previously used in other publications [7-9, 25].

Fig. S3

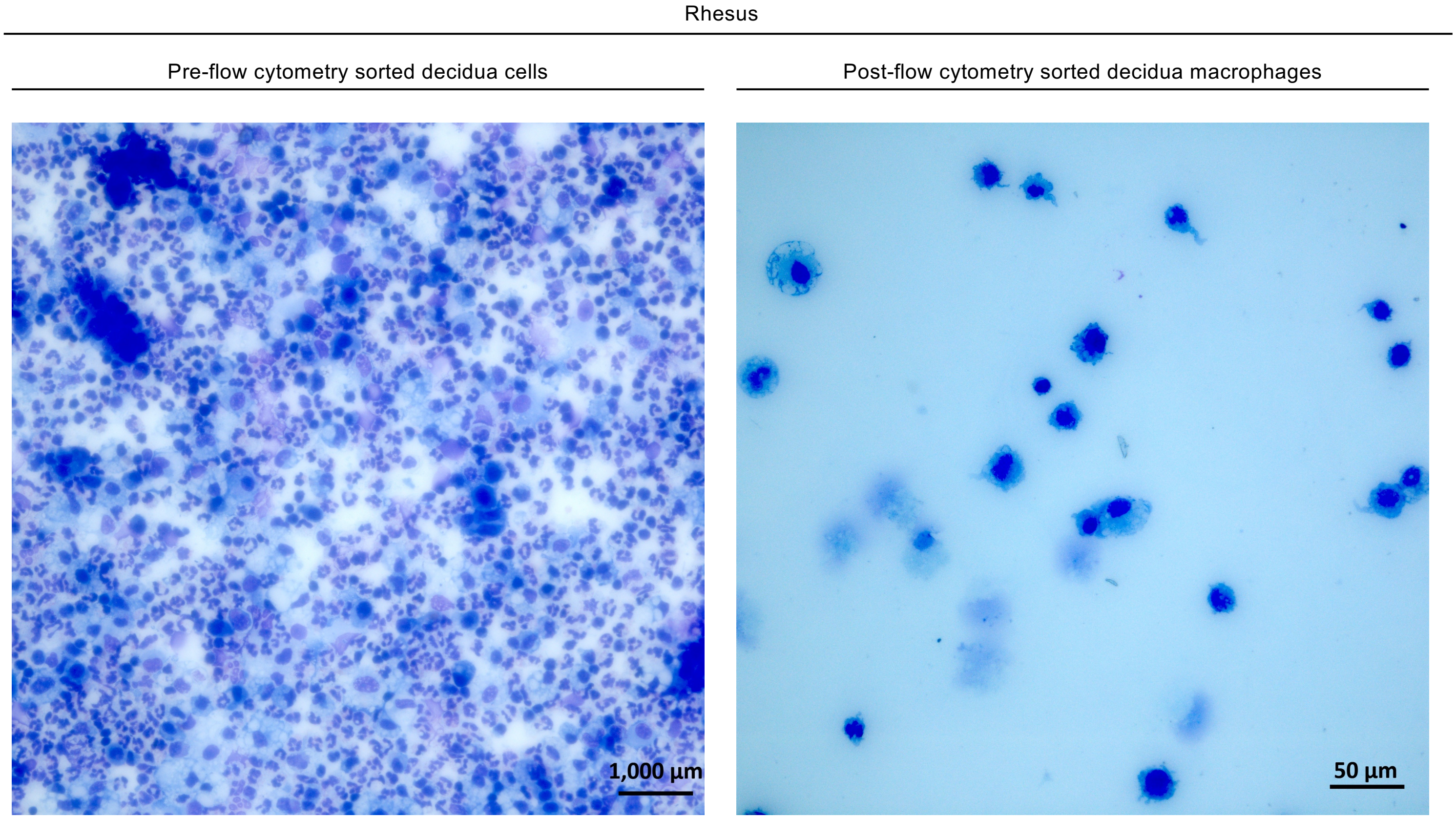

Fig. S3. Purity of decidua macrophages (DMs) after flow-sorting. Rhesus DMs (defined as Live cells+CD45^{+}CD3^{-}CD19^{-}CD20^{-}CD56^{-} CD14^{high}CD88^{+}HLA-DR^{+} cells) were sorted by FACS from the three groups of animals with a purity >98% based on morphology. Representative (n=3) Diff-Quick staining of pre- and post-sorted cells. The integrity of purified total flow cytometry-sorted DMs (10-50 ng per sample) was assessed using HighSensitivity RNA ScreenTapes on the TapeStation 2200 (Agilent Technologies).

Fig. S4

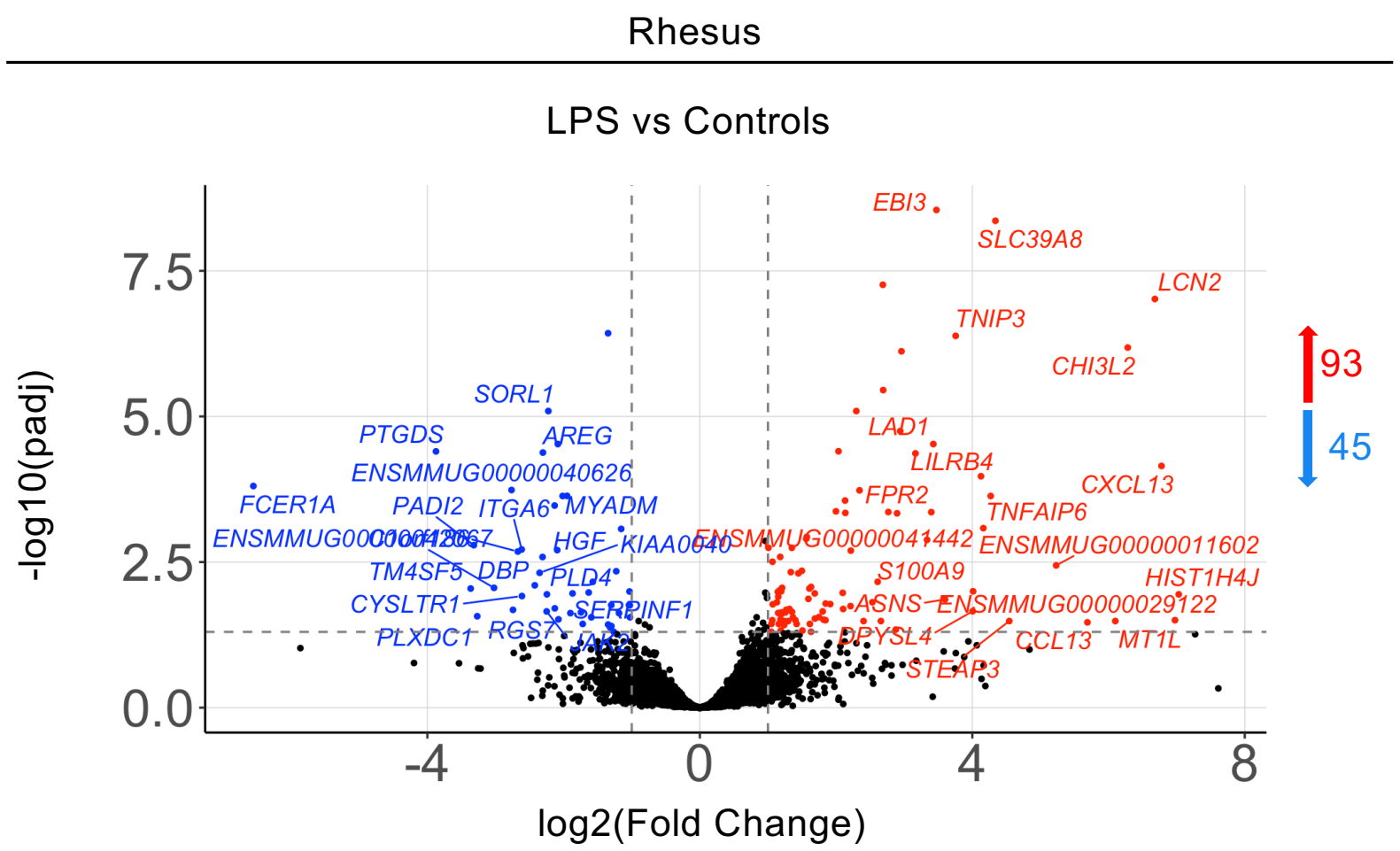

Fig. S4. LPS exposure upregulates 93 genes and downregulates 45 genes compared to Controls. The volcano plot displays differentially expressed genes (DEGs) in Rhesus LPS vs Controls. Red and blue dots indicate respectively upregulated and downregulated genes with an FDR adjusted p value < 0.05 and DEG > 1-fold change.

Fig. S5

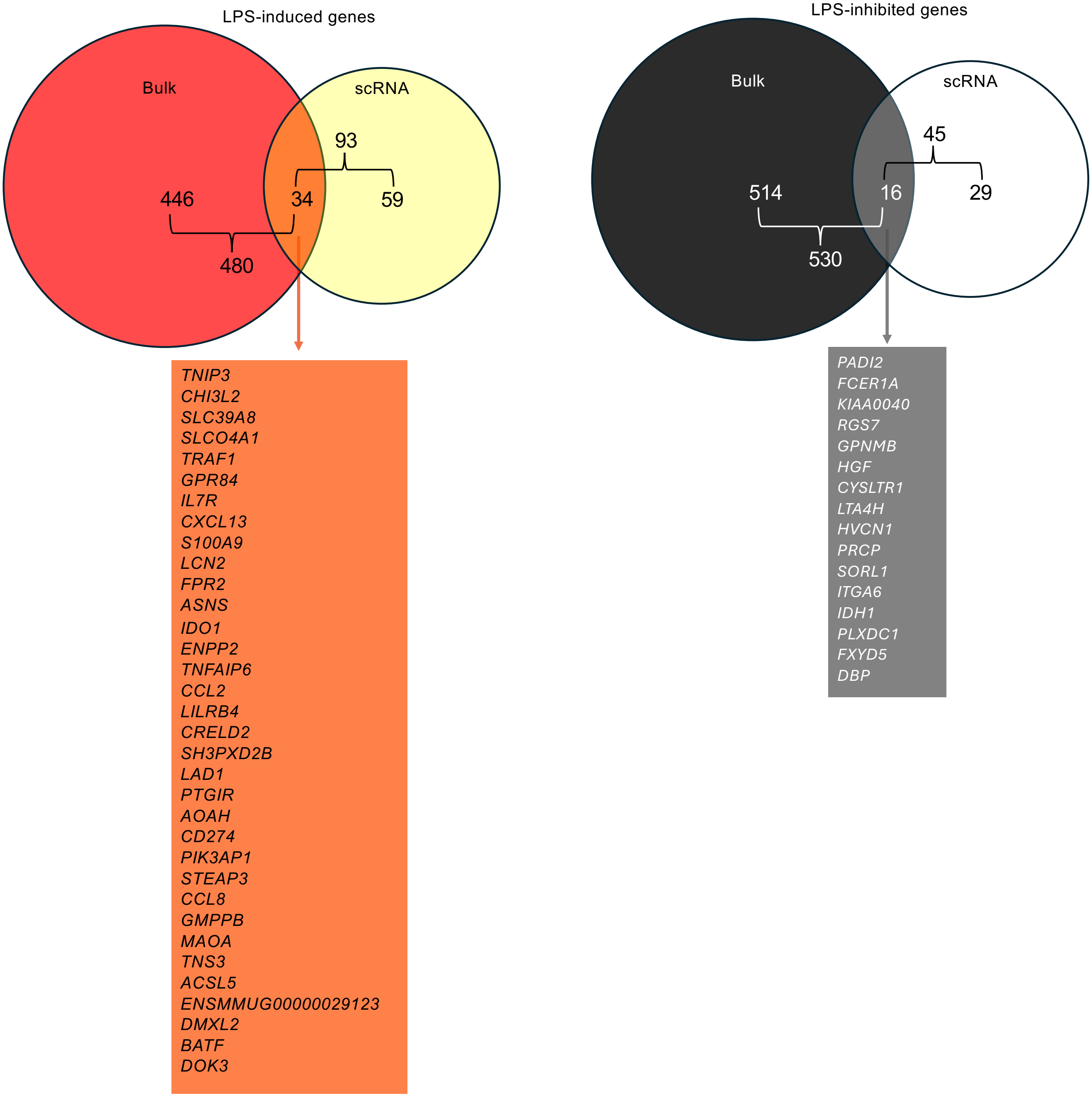

Fig. S5. Bulk RNAseq and scRNAseq analyses identified 34 commonly upregulated and 16 commonly downregulated shared in Rhesus DMs. Notably, these overlapping gene sets include both pro-inflammatory and anti-inflammatory regulators, underscoring a mixed M1/M2 polarization signature in DMs.

Fig. S6

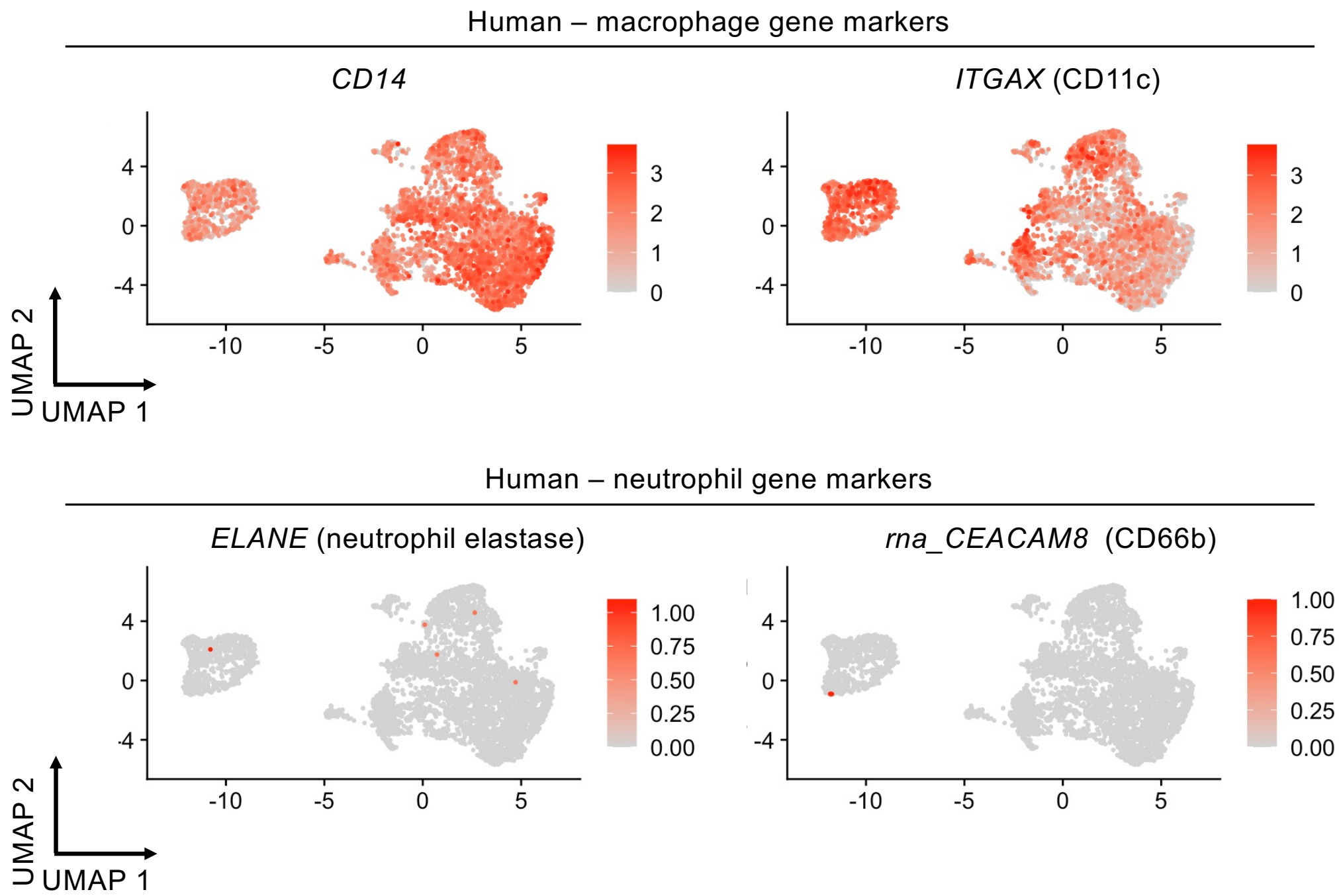

Fig. S6. Human cluster 1 does not express neutrophil marker genes. UMAP graphs showing two representative gene markers expressed by macrophages (*CD14* and *ITGAX*) and by neutrophils (*ELANE* and *CEACAM8*).

Fig. S7

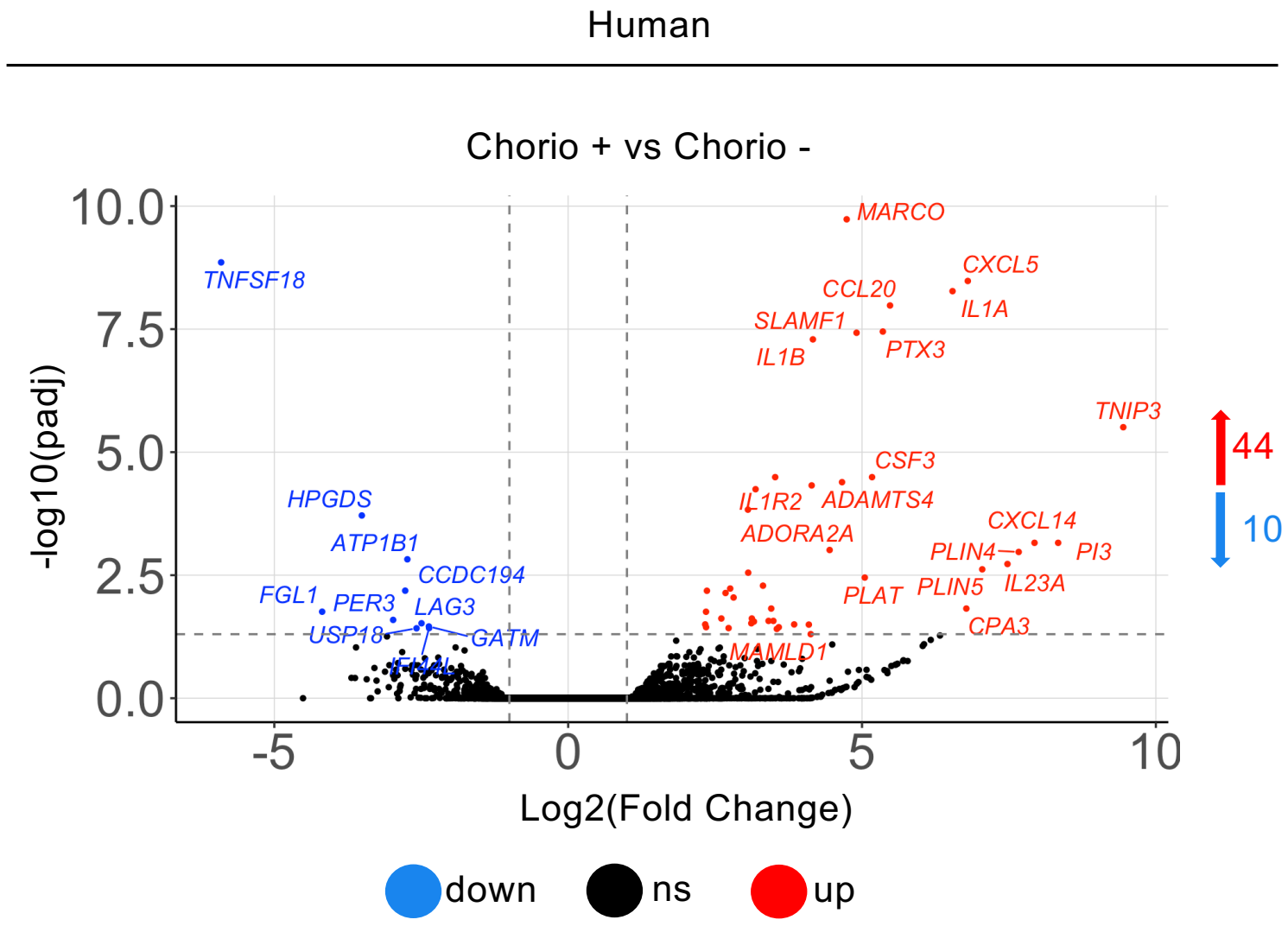

Fig. S7. Chorioamnionitis upregulates 44 genes and downregulates 10 genes compared to samples without chorioamnionitis. The volcano plots displays differentially expressed genes (DEGs) in human Chorio + vs Chorio - DMs. Red and blue dots indicate respectively upregulated and downregulated genes with an FDR adjusted p value < 0.05 and DEG > 1-fold change.

Fig. S8

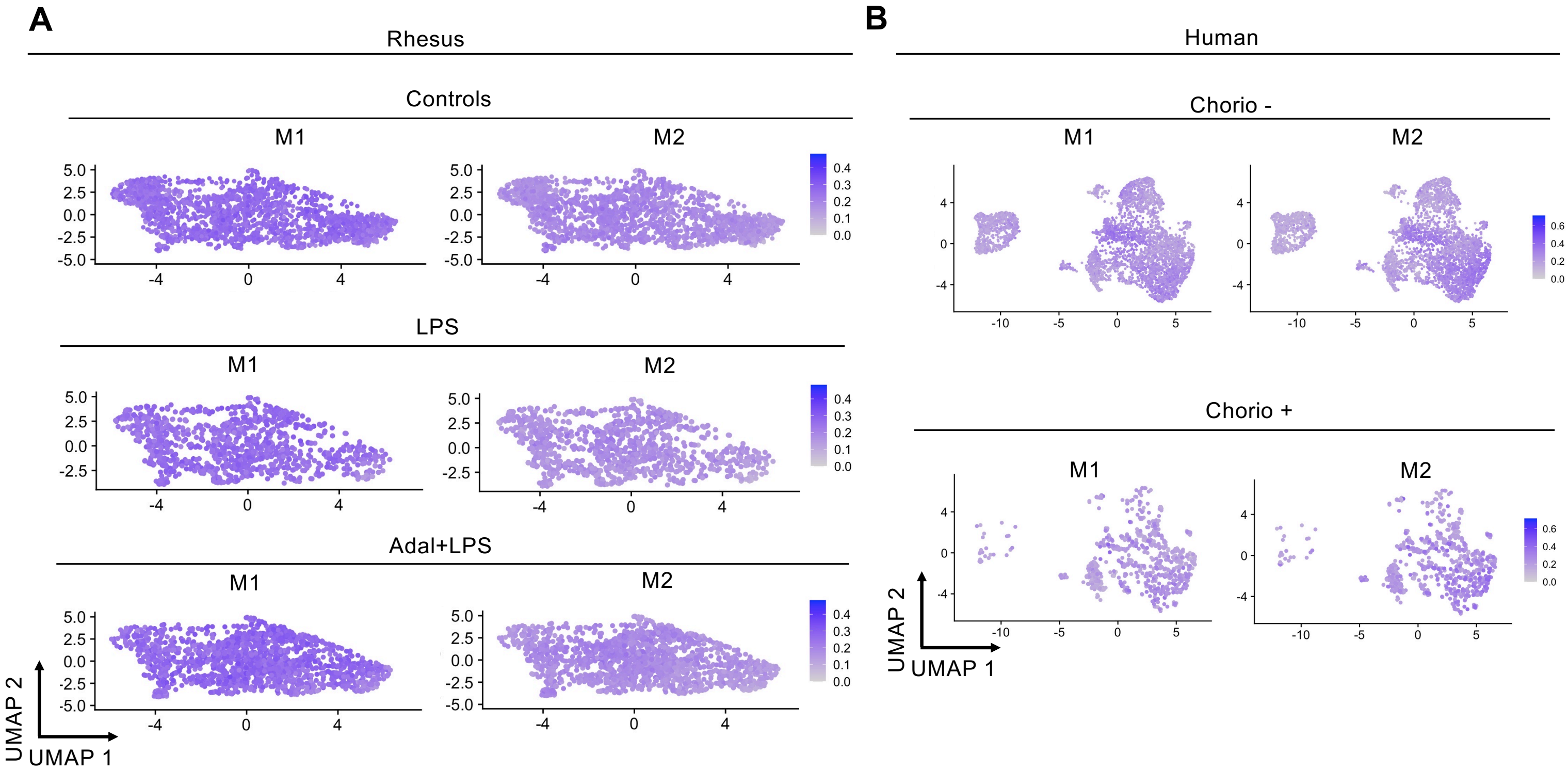

Fig. S8. UMAP visualizations illustrated the expression of M1 and M2 marker genes in DMs from both Rhesus and humans. **A** In Rhesus DMs, cells consistently co-express pro-inflammatory M1 markers and anti-inflammatory M2 markers, independent of treatment conditions. **B** Human DMs exhibit a similar mixed M1/M2 signature, reinforcing the presence of a conserved hybrid polarization state across species.

Fig. S9

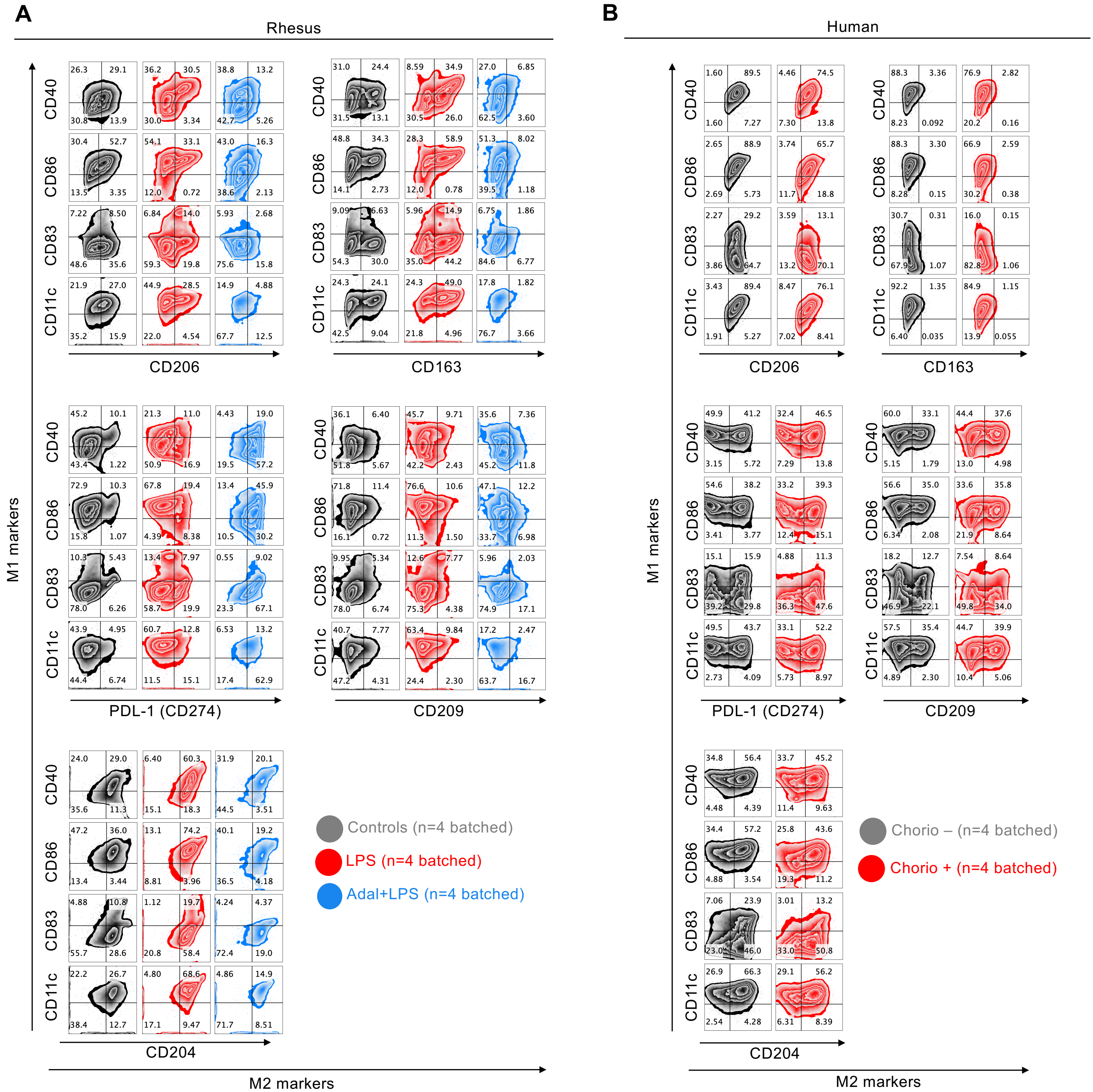

Fig. S9. DMs simultaneously express markers associated with both M1 and M2 phenotypes regardless of chorioamnionitis. **A-B** Flow cytometry of decidua cell suspensions stained with a cocktail of M1 (CD11c, CD83, CD86, and CD40) and M2 (CD206, CD163, PDL-1[CD274], CD209, and CD204) markers. A uniform gating strategy was applied to four samples belonging to each group for both Rhesus and human using the batch processing function in Flowjo. Contour plots gated on DMs show the co-expression of M1 and M2 markers that do not change upon chorioamnionitis in both Rhesus and human.

Fig. S10

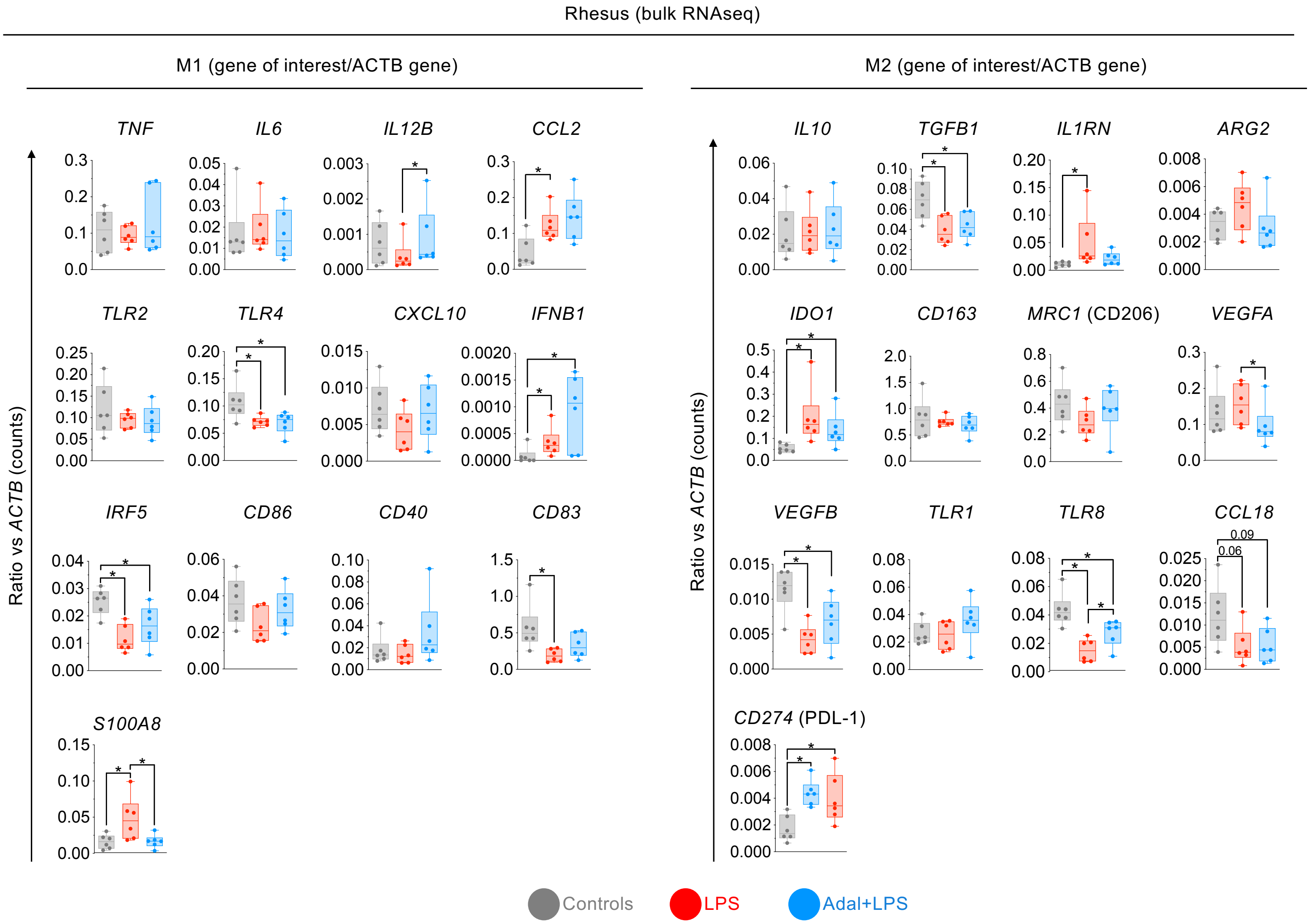

Fig. S10. Normalized counts of gene expression from bulk RNAseq of flow sorted Rhesus DMs. Representative genes values are normalized to *ACTB* gene (actin B) (Controls n=6; LPS n=6; Adal+LPS n=6 for all the genes). Data are mean, SEM, *p<0.05 between comparators (Mann–Whitney U test).

Fig. S11

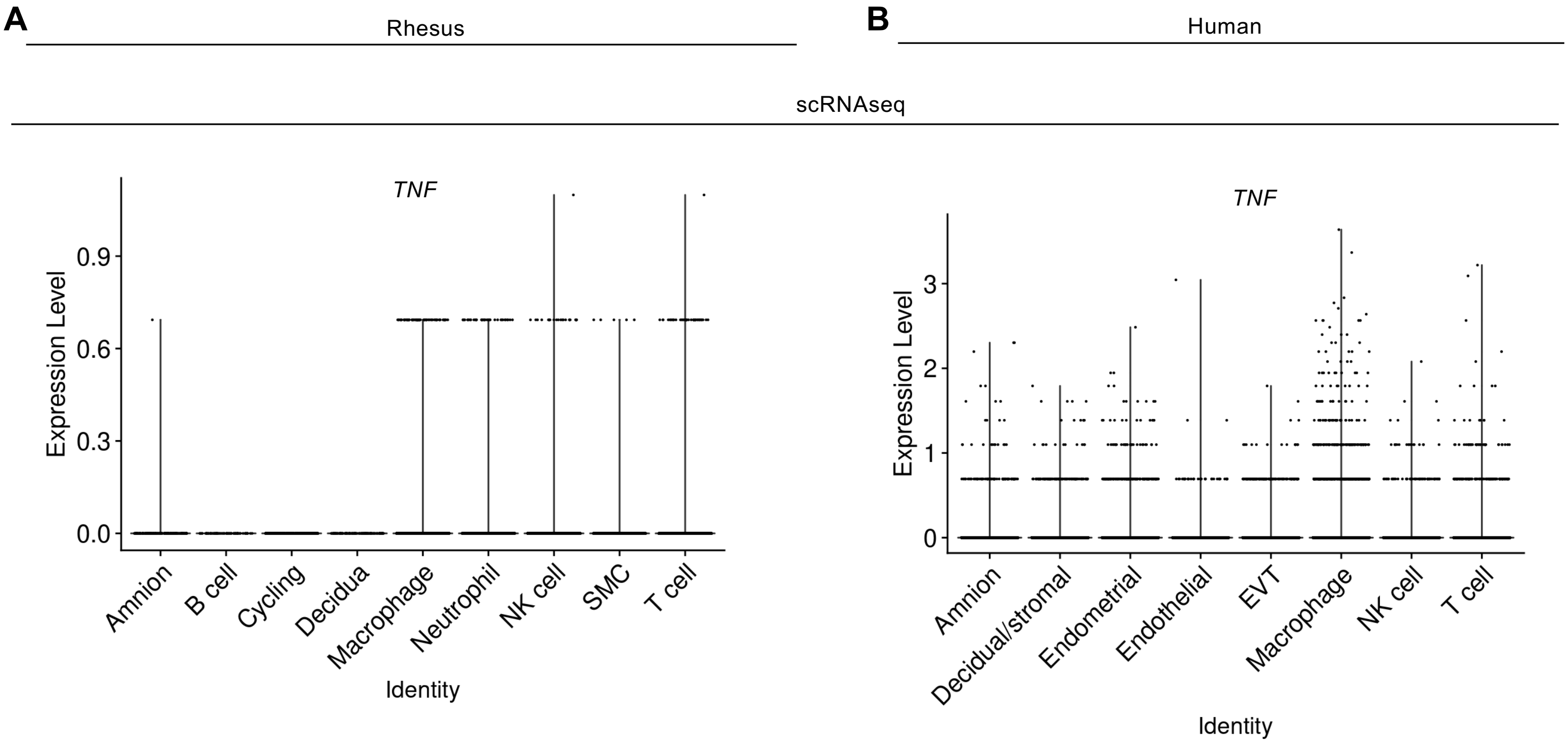

Fig. S11. Minimal *TNF* mRNA expression. Violin plot showed *TNF* expression in **A** Rhesus and **B** human decidua cell types.

Fig. S12

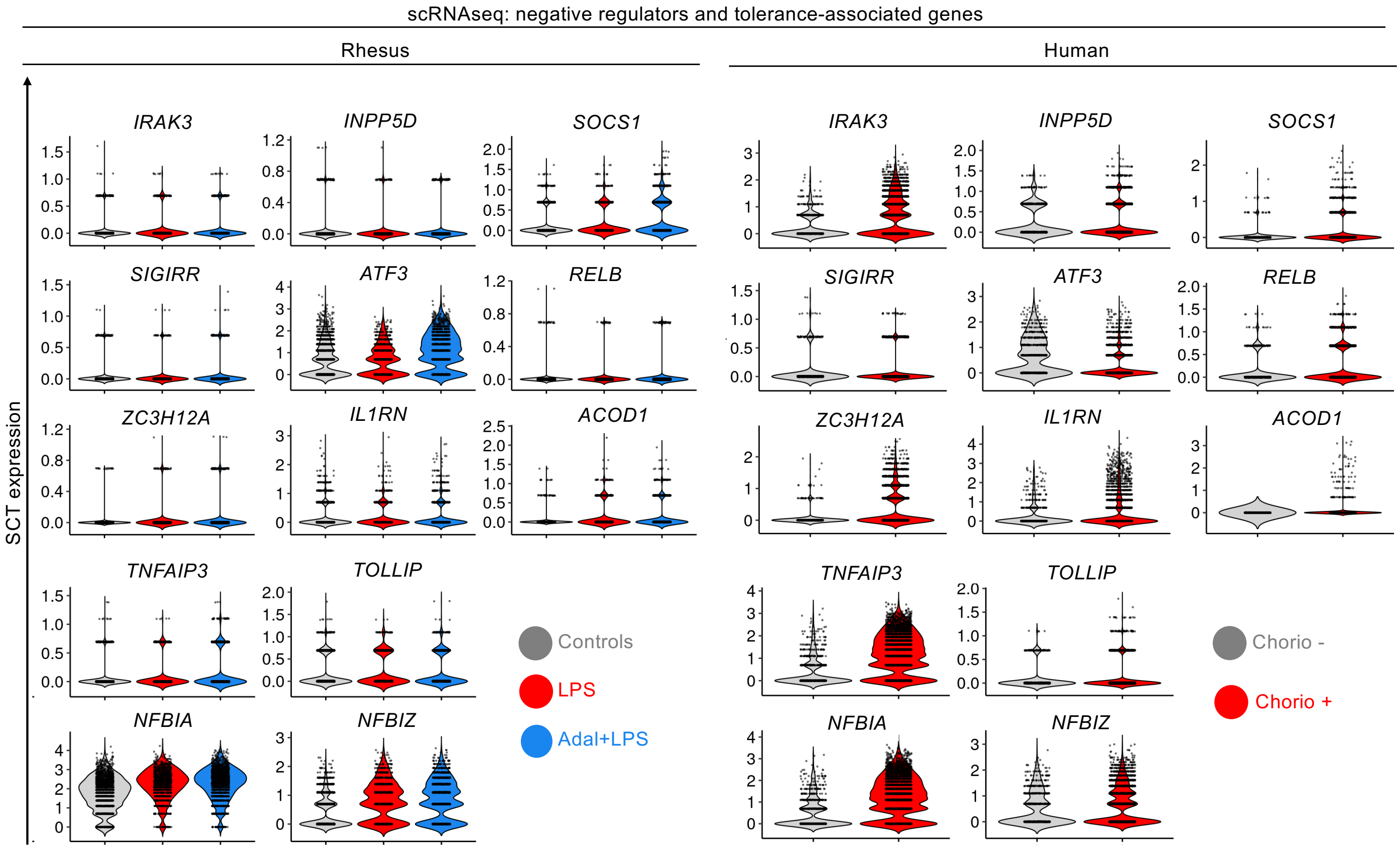

Fig. S12. Violin plot show the expression of selected negative regulators and tolerance-associated genes in Rhesus and human DMs.

Fig. S13

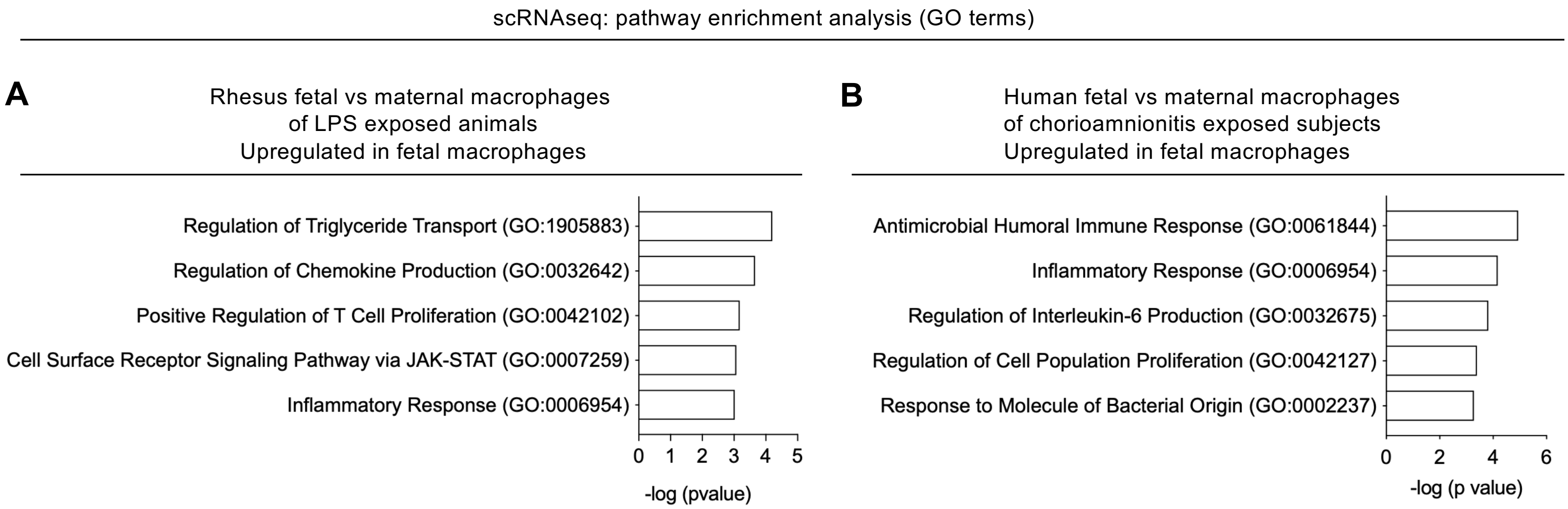

Fig. S13. **A** Top 5 GO terms of **A** 51 differentially expressed genes of LPS exposed fetal macrophages compared to maternal macrophages and **B** 47 differentially expressed genes of chorioamnionitis exposed fetal macrophages compared to maternal macrophages (FDR adjusted p value ≤ 0.05, FC 2 (log2FC 1)).

Supplementary Methods

Decidua Macrophage isolation and bulk RNAseq

Dispase II (Life Technologies, Grand Island, NY) and collagenase A (Roche, Indianapolis, IN) followed by DNase I (Roche) were used to digest decidua cells after washing. Decidua macrophages (DMs) were purified from decidua cell suspension by FACSAria Cell Sorter (BD Bioscience), using the same gating strategy as described for immunophenotyping.[7,8] Sorted DM purity was >97% determined by Diff-Quick Staining (Fig. S3). Total RNA was extracted from 1.5-3.5 x 10^6 sorted DMs after adding TRIzol (ThermofisherScientific) and then using Direct-Zol RNA MicroPrep kit (ZYMO Research, Irvine, CA) for extracting small quantities of RNA. The integrity of purified total RNA from sorted DMs was assessed using HighSensitivity RNA ScreenTapes on the TapeStation 2200 (Agilent Technologies). Two hundred to three hundred nanograms of material were used for the NEBNext rRNA Depletion kit (cat# E6350). Next, RNA libraries were created using the NEBNext Ultra II RNA kit (cat# E7765). Two hundred ng of each final library was used for capture hybridization using Illumina TruSeq Exome, (cat #20020490) and Illumina Exome Panel (cat #20020183) according to the manufacturer's instructions. DNA D1000 Assay (TapeStation 2200 - Agilent Technologies) and quantified using a Qubit dsDNA BR Assay (Life Technologies) were used for quality control for the final libraries. Diluted libraries were combined and sequencing was done 50 single-end on a HiSeq3000 (Illumina). Reads were mapped with STAR 2.5.3a to the Macaca mulatta genome (Mmul 8.0.1). Counts per gene were determined using the options –quantMode GeneCounts. DESeq2 was used for differential expression analysis. The normalized counts were obtained from the DESeq2 analysis. Principal Component Analysis (PCA) was performed with the

plotPCA function in DESeq2 after regularized log transformation. Heatmaps were plotted on the log2 value of the normalized counts. GO term and KEGG pathway inferences were generated using Enrichr.

Single-cell (sc)RNAseq: Library construction, sequencing and analysis

Eight Rhesus amnion samples (ctrl n=2; LPS n=3; Adal+LPS n=3) were used.[9,10] Of note, the majority of Rhesus samples used for scRNAseq analysis were also used for bulk RNAseq analysis (Table S1B). First, Rhesus fresh chorioamnion-decidua (CAMD) cells were stained to Sytox Green and sorted to exclude dead cells (FACSAria, BD Biosciences). Live, unseparated CAMD were then microfluidically separated, barcoded and 10X Single Cell 30 v2 Reagent Kits were used to create scRNA seq libraries. Single-cell Gel Bead-In- EMulsions (GEMs) were generated by loading single cell suspensions onto a Chromium Controller instrument. GEM-RT were done in a Veriti 96-well thermal cycler (Thermo Fisher Scientific, Waltham, MA), GEMs were harvested and the cDNAs were amplified and cleaned with SPRIselect Reagent Kit. Indexed sequencing libraries were created using Chromium Single-Cell 30 Library Kit for A-tailing, enzymatic fragmentation, adapter ligation, end-repair, ligation cleanup, sample index PCR, and PCR cleanup. KAPA Library Quantification Kit (KAPA Biosystems, Wilmington, MA) was used to quantify the sequenced libraries. A custom sequencing setting (26 bp for Read 1 and 91 bp for Read 2) was used to load sequenced libraries onto NovaSeq2 (Illumina, San Diego, CA). For human samples, CAMD cells were isolated from FFPE tissue sections for use with Chromium Fixed RNA Profiling kit (10X Genomics), according to manufacturer's instructions, as previously descripbed (Cell Reports, 2025). Briefly, four human FFPE CAMD blocks (Chorio + n=2 and Chorio - n=2) were sectioned at 25 μM. 2

sections/subject were transferred to gentleMACS C tube (Miltenyi Biotech) keeping the scroll intact. Sections were deparaffinized with xylene/ethanol and then transferred to gentleMACS Octo (program 37C_FFPE_1) in dissociation enzyme mix (Liberase TH – Millipore Sigma). Pellet was filtered through a 30 µM strainer (Miltenyi Biotech), washed in PBS, and cells were stained with Ethidium Homodimer-1 and counted with a Countess II FL Automated Cell Counter (ThermoFisher Scientific). A multiplexed library was constructed using the 4BC Human Transcriptome Chromium Fixed RNA Profiling Kit (PN-1000475; 10X Genomics) according to the manufacturer's protocol, according to the following specifications: Probes were incubated with single cell suspensions for 20 h at 42° C. Eleven cycles were used for the sample index PCR. The multiplexed library was sequenced on the NovaSeq6000 using the NovaSeq S1 100 cycle flowcell. Data processing including read alignment (to the reference genome Mmul 8.0.1 (Rhesus) and GRCh38 (Human)), quality control, and gene quantification was accomplished using 10X Cell Ranger software (version 2.1.1). Using the cellranger aggr pipeline, the samples were merged into a single expression matrix. The cells were clustered into a merged matrix using the R package Seurat (v5.3.0). Cells with more than 5% of mitochondrial expression or less than 100 unique genes (nFeature_RNA), or less than 500 total transcripts (nCount_RNA), were filtered out as low-quality cells.

Following initial preprocessing and quality control, doublets were identified and removed using DoubletFinder. Seurat objects were split by sample (orig.ident) to allow batch-aware normalization. Each assay was normalized using SCTransform v2, regressing out mitochondrial content. Cell cycle scoring was performed using S and G2/M phase gene sets obtained from published markers and annotated via

AnnotationHub; however, cell cycle variables were not regressed during normalization as no significant cell cycle-driven bias was observed. Principal component analysis (PCA) was performed on scaled residuals from SCTransform, and the number of significant principal components was determined using combined criteria of cumulative variance, percent variance per PC, and change in consecutive PC variance. Cells were clustered based on significant PCs using FindNeighbors and FindClusters and visualized in two-dimensional space using UMAP. Batch effects across samples were corrected using Harmony, and clustering and UMAP were recalculated on the integrated embeddings. To prepare the dataset for differential expression analyses, counts from multiple SCTransform models were reconciled using PrepSCTFindMarkers, which recalculates normalized counts across all batches. Finally, layers were joined to generate a unified assay, which was used for downstream analyses, including cell type identification, visualization, and marker gene analysis.

For macrophage-specific analyses, subsets of macrophages were extracted from the full Seurat objects using cell type annotation[9-13]. The same preprocessing, normalization, cell cycle scoring, PCA, clustering, Harmony integration, and UMAP visualization workflow was applied to these macrophage subsets. Following integration, SCT models were merged using PrepSCTFindMarkers, and layers were joined to generate the final unified macrophage assay used for all downstream macrophage analyses.

Cluster-specific marker genes for macrophages were identified by computing differential expression for each macrophage cluster using FindMarkers on SCT residuals. For each cluster, markers were filtered for adjusted p-value < 0.05, pct.1 > 0.3, and

log2FC > 1; the top 10 markers per cluster were selected, combined across all clusters while preserving ordering, and used to generate a bubble plot summarizing average expression and percent expression per cluster.

To evaluate macrophage polarization, UCell signatures corresponding to M1 and M2 polarization programs were computed at single-cell resolution using AddModuleScore_UCell. Scores were mapped onto UMAP embeddings using FeaturePlot. For condition-specific comparisons, macrophage subsets (Rhesus: Ctrl, LPS, Adal+LPS; Human: Chorio +, Chorio neg) were processed separately, M1/M2 signatures were calculated, and smoothed signature distributions were computed using UCell function SmoothKNN on Harmony embeddings.

Differences in macrophage cluster proportions across conditions were assessed using the scProportionTest R package. Statistical significance was determined through a permutation-based test to calculate p-values for each cluster, which were subsequently adjusted for multiple comparisons using the Benjamini-Hochberg (FDR) method. Additionally, 95% confidence intervals for the magnitude of difference (Log2 Fold Change) were estimated via bootstrapping. Clusters were considered significant if they met the dual criteria of an $FDR < 0.05$ and an $|Log2FC| > 0.58$. Results were visualized using permutation plots.

To identify fetal macrophages, Y chromosome–linked genes (ASMTY, TSPY, IL3RAY, SRY, ZFY, PRKY, AMELY, ANT3Y, AZF2, BPY2, AZF1, DAZ, RBM1, RBM2, UTY, USP9Y, RPS4Y1) were used as a fetal marker set. Cells expressing at least one Y-linked gene with SCT-normalized expression > 0 were classified as fetal macrophages. In the Rhesus macaque dataset, applying this criterion resulted in the detection of

RPS4Y1 as the only Y-linked gene expressed, whereas in the human dataset the detected Y-linked genes were ZFY, PRKY, UTY, and USP9Y.

For pseudobulk transcriptomic analysis, pseudobulk count matrices were generated by aggregating RNA assay counts per sample and per macrophage subtype using AggregateExpression. After grouping by condition and reinserting sample-level metadata, pseudobulk differential expression analyses were performed using DESeq2. For human decidua macrophages, samples were grouped by condition (Chorio + vs Chorio -) and tested using DESeqDataSetFromMatrix followed by DESeq. For Rhesus decidua macrophages, pseudobulk counts were generated across Ctrl, LPS, and Adal+LPS conditions; DESeq2 models were fit with condition as design, and contrasts (LPS vs Ctrl, Adal+LPS vs LPS, Adal+LPS vs Ctrl) were computed. Only genes with at least 10 counts in ≥2 samples were retained. Differential expression results were visualized as volcano plots using ggplot2. Genes meeting significance thresholds (adjusted p-value < 0.05 and $|log2FC| > 1$) were highlighted.

Functional pathway analysis and Upstream Regulator analysis were performed using the Ingenuity Pathway Analysis (IPA) software. Differentially Expressed Genes (DEGs) used as input for the IPA analysis were selected based on a significance threshold of padj <0.05 and $|log2\ FC|>1$ and were divided according to the following comparisons: human Macrophages (Chorio + vs Chorio -): DEGs previously identified via pseudobulk analysis on the scRNAseq data were used. Rhesus decidua macrophages (LPS vs Ctrl and Adal+LPS vs LPS): DEGs identified from Bulk RNA-sequencing (traditional RNA-seq) data were used to increase statistical power and gene coverage for these specific comparisons. For each supplied set of differentially expressed genes, core

analyses were executed within IPA, including Canonical Pathways to detect activated or inhibited signaling and biochemical routes, and Upstream Regulators to predict transcription factors and signaling molecules that explain the observed changes in gene expression.

Cluster-specific marker genes were identified using the FindAllMarkers function in Seurat (Wilcoxon rank-sum test, only.pos = TRUE, min.pct = 0.3, logfc.threshold = 1) and filtered for significance at an adjusted p-value < 0.05 (Bonferroni correction). To assess transcriptional similarity between human and Rhesus macrophages, marker genes of each Rhesus cluster (excluding genes lacking annotation) were used to define cluster-specific gene signatures. UCell scores were computed for each human macrophage using these signatures and smoothened by SmoothKNN on Harmony embeddings. The median UCell score across all cells in a human cluster was used as the cluster-level similarity score.

To compare transcriptional responses between species in response to LPS stimulation, log2 fold changes (log2FC) obtained from the pseudobulk differential expression analysis in Rhesus macaque were plotted against the corresponding log2FC values in human. Each point in the scatter plot represents a gene, with the x-axis showing log2FC in rhesus macaque and the y-axis showing log2FC in human. Genes were categorized based on statistical significance in each species and color-coded accordingly: genes significantly differentially expressed in both species, only in human, or only in rhesus macaque. Spearman correlation analysis revealed a moderate correlation between rhesus macaque and human log2FC values.

Flow cytometry

For immunophenotyping and functional studies, the samples were analyzed by LSR Fortessa 2, BD Biosciences. For immunophenotyping, decidua cells were treated with 20 μg/ml human IgG to block Fc receptors, stained for surface markers for 30 minutes at 4°C in PBS, washed, and fixed in fixative stabilizing buffer (BD Bioscience).

For *ex vivo* intracellular detection of TNFα, decidua cells isolated immediately post-delivery without exogenous stimulation were first stained for surface markers, fixed and permeabilized with Cytofix/Cytoperm (BD Biosciences) and stained for intracellular TNFα mAb. Cells were washed and fixed in fixative stabilizing buffer (BD Bioscience).

For *in vitro* intracellular detection of TNFα, decidua cells were thawed and 0.5x10^6/ml decidua cells were cultured overnight in 24-well plate in DMEM 10% FCS (Thermofisher) without stimulation to allow recovery after thawing. Next, the decidua cells were stimulated with or without 100ng/ml LPS (Sigma-Aldrich, St. Louis, MO) for 3 hours. Golgi Stop (BD Bioscience) was added to all cells for the last two hours. Cells were first stained for surface markers, fixed and permeabilized with Cytofix/Cytoperm (BD Biosciences) and stained for intracellular TNFα mAb. Cells were washed and fixed in fixative stabilizing buffer (BD Bioscience). LPS dose-response and time-course parameters were optimized in preceding assays to maximize TNFα production.

At least 500,000 events were recorded for each sample. Doublets were excluded based on forward scatter properties, and dead cells were excluded using LIVE/DEAD Fixable dead cell stain (Invitrogen). Unstained, isotype control, or negative biological population were used to determine positive staining for each marker.

FACS

For bulk RNAseq, we used purified decidua macrophages. Decidua cells were stained as described above but not fixed in fixative stabilizing buffer. Cells were resuspended in PBS+2%FCS and decidua macrophages were sorted using the FACSAria Cell Sorter (BD Bioscience). Purity of sorted decidua macrophages (>97%) was determined by Diff-Quick Staining (Fig. S3).

For scRNAseq experiment with Rhesus samples, $5x10^6$ CAMD decidua cells were stained with Styox Green (Thermo Fisher Scientific) as per manufacturer's instructions, resuspended in PBS+2%FCS and live cells were sorted using the FACSAria Cell Sorter (BD Bioscience).
